# Switchable selectivity in Pd-catalyzed [3 + 2] annulations of γ-oxy-2-cycloalkenones with 3-oxoglutarates: C–C/C–C vs C–C/O–C bond formation

**DOI:** 10.3762/bjoc.15.107

**Published:** 2019-05-16

**Authors:** Yang Liu, Julie Oble, Giovanni Poli

**Affiliations:** 1 Sorbonne Université, Faculté des Sciences et Ingénierie, CNRS, Institut Parisien de Chimie Moléculaire, IPCM, 4 place Jussieu, 75005 Paris, France

**Keywords:** 1,4-addition, annulation, decarboxylation, palladium, Pd-catalyzed allylation reaction

## Abstract

Two complementary [3 + 2] annulation protocols between 3-oxoglutarates and cyclic γ-oxy-2-cycloalkenones, simply differing on the reaction temperature, are disclosed. These domino transformations allow C–C/O–C or C–C/C–C [3 + 2] annulations at will, via an intermolecular Pd-catalyzed *C*-allylation/intramolecular *O*- or *C*-1,4-addition sequence, respectively. In particular, exploiting the reversibility of the *O*-1,4-addition step, in combination with the irreversible *C*-1,4-addition/decarboxylation path, the intramolecular conjugate addition step could be diverted from the kinetic (*O*-alkylation) to the thermodynamic path (*C*-alkylation) thanks to a simple temperature increase. Crucial for the success of this bis-nucleophile/bis-electrophile [3 + 2] annulation is its well-defined step chronology in combination with the total chemoselectivity of the former step. This [3 + 2] C–C/O–C bond forming annulation protocol could be also extended to 1,3,5-triketones as well as 1,3-bis-sulfonylpropan-2-one bis-nucleophiles.

## Introduction

The development of new strategies for the synthesis of complex carbocyclic and heterocyclic structures remains a general topic for the synthetic chemists [1]. In the past decades, palladium chemistry has gained an important place in the toolbox of chemists, and its use became a privileged strategy for the selective formation of carbon–carbon and carbon–heteroatom bonds [2–5]. Among the different types of palladium-catalyzed transformations, domino – alias cascade – transformations [6–11] as well as annulation reactions [12–15] occupy a special place as they facilitate the synthesis of a variety of complex cycles in a single synthetic operation, through sequential and mechanistically independent bond-forming steps.

**Scheme 1:**
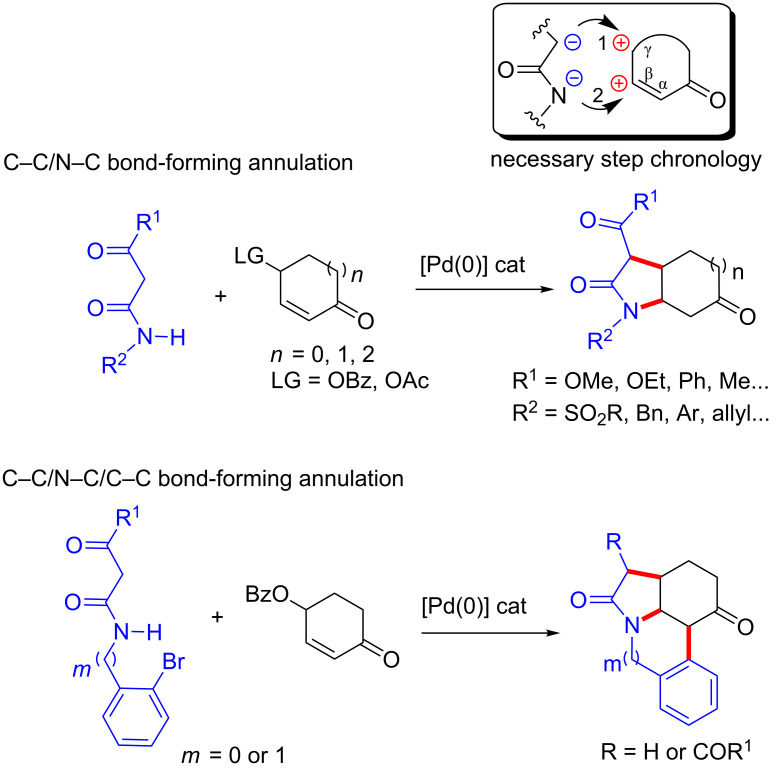
Previously developed bis-nucleophile/bis-electrophile [3 + 2] annulations.

In the frame of our long-term project dedicated to domino sequences [16–22] and Pd-catalyzed transformations [23–30], we recently reported a Pd(0)-catalyzed synthesis of bi- and tricyclic structures incorporating pyrrolidone rings (Scheme 1) [31]. In this transformation, resonance-stabilized acetamides and cyclic α,β-unsaturated-γ-oxicarbonyl derivatives are used as bis-nucleophile and bis-electrophile partners, respectively.

This process involves an intermolecular Pd(0)-catalyzed *C*-allylation (Tsuji–Trost reaction)/intramolecular nitrogen 1,4-addition sequence (Scheme 1, top reaction). The success of this bis-nucleophile/bis-electrophile [3 + 2] C–C/N–C bond-forming annulation is due to the well-defined chronology of the steps and the total chemoselectivity of the initial step (*C*-allylation). Another non-trivial feature of this process is that the possible undesired stoichiometric intermolecular 1,4-addition step (i.e., the potential initial addition of the nucleophile to the C-β of the bis-electrophile) has to be slower than the intermolecular addition of the nucleophile to the catalytically generated η^3^-allylpalladium complex, or it has to be at least a reversible process [32]. Furthermore, when the newly formed annulation product contains an *ortho*-haloaryl moiety at the nitrogen substituent, an additional intramolecular keto α-arylation step can be involved in the cascade, thereby forming two new cycles and three new bonds in the same synthetic operation (Scheme 1, bottom reaction).

**Scheme 2:**
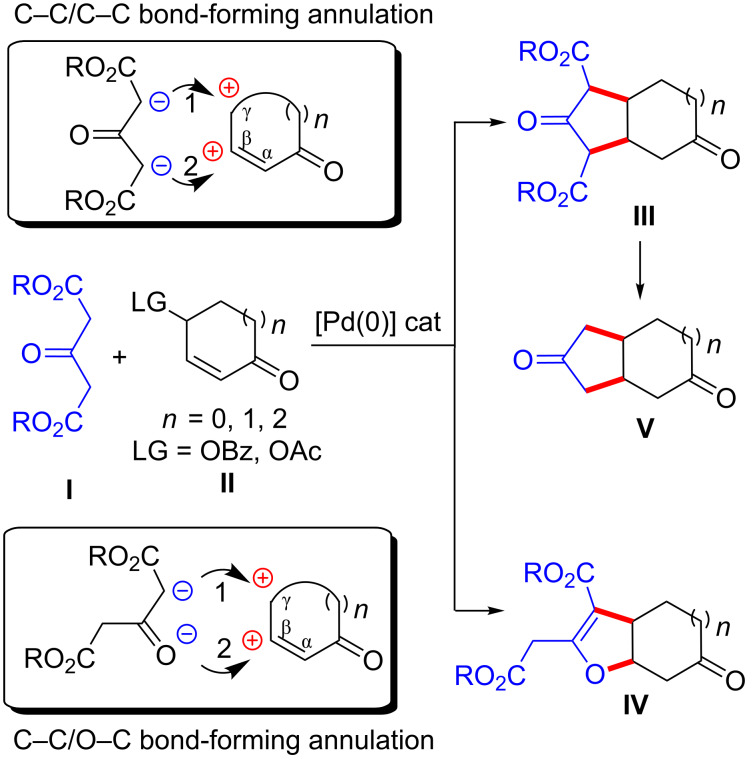
Concept: [3 + 2] C–C/C–C vs C–C/O–C bond-forming annulations.

**Figure 1:**
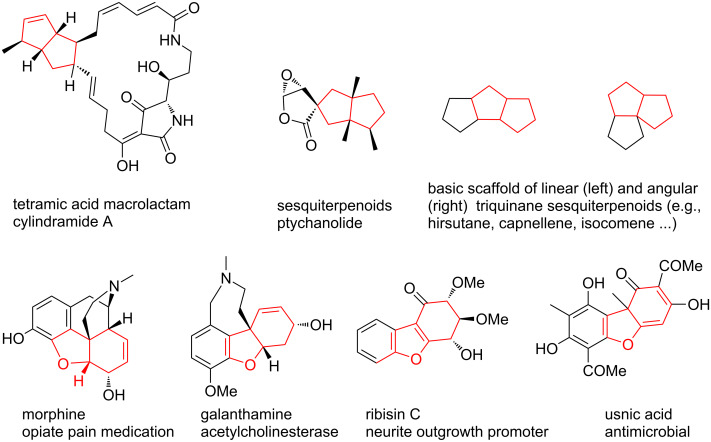
Examples of annulated cylopentanic (top) and furan-based (bottom) substructures in natural products.

We next decided to extend the scope of this strategy to dialkyl-3-oxoglutarates **I** as the bis-nucleophile partners [33] in the reaction with cyclic α,β-unsaturated-γ-oxycarbonyl derivatives **II** as the bis-electrophiles (Scheme 2). Interestingly, this new bis-nucleophile/bis-electrophile combination may allow direct access to either fused bicyclic cyclopentanic (pentalene-, indene-, or azulene-type) structures via a γ-*C*-allylation/β-*C*-1,4 addition process, or annulated furan-based motifs through a γ-*C*-allylation/β-*O*-1,4 addition process, both motifs being incorporated into biologically relevant pharmaceuticals and/or natural products (Figure 1) [34–38]. Indeed, we anticipated that the latter step might occur through either *C*-addition or *O*-addition (Scheme 2, products **III** (or **V**) vs **IV**) [32,39–42]. Therefore, an inherent challenge associated to this ambident nucleophile is chemoselectivity control.

Herein we disclose two chemodivergent [3 + 2] annulation reactions taking place between dialkyl-3-oxoglutarates **I** and α,β-unsaturated-γ-oxycarbonyl derivatives **II** that differ simply by the reaction temperature adopted. These methods allow the exclusive [3 + 2] C–C/C–C or C–C/O–C annulation at will, thus providing an easy access to annulated cyclopentanic structures **III** or annulated furan-based motifs **IV**, respectively. Additionally, in the case of C–C/C–C bond forming annulations, the products **III** can undergo two decarboxylation steps leading to bis-cycloalkanone derivatives **V**.

## Results and Discussion

### Optimization

We started our investigation using dimethyl 3-oxoglutarate (**1a**) and 2-cyclohexenone 4-benzoate (**2a**) [43–44] as bis-nucleophile and bis-electrophile model substrates, respectively (Table 1). In line with our previously developed standard reaction conditions for [3 + 2] C–C/N–C bond-forming annulation [31], the first tests were performed with the following two catalytic systems: [Pd(η^3^-C_3_H_5_)Cl]_2_ (5 mol %), dppf (15 mol %)], and [Pd(OAc)_2_ (10 mol %), dppb (15 mol %)] in THF at room temperature (Table 1, entries 1 and 2). These conditions promptly (ca. 1 hour) generated annulated product **4a** arising from *C*-allylation (C–C bond forming)/intramolecular *O*-Michael addition (O–C bond forming) sequence. The purification of the crude reaction was easier using the Pd(OAc)_2_ and dppb system (Table 1, entry 2), which was thus chosen to continue the optimization. The effect of the solvent was then assessed. While no reaction took place in CH_3_CN (Table 1, entry 3), DMF (Table 1, entry 4) and DMSO (Table 1, entry 5) allowed the formation of **4a** in 66% and 75% yield, respectively. The use of another bidentate phosphine such as dppe gave no improvement (Table 1, entry 6), showing only traces of **4a**. The influence of the temperature in DMSO was examined next. The reaction performed at 75 °C gave a similar yield for compound **4a** (compare entries 5 and 7 in Table 1), whereas the reaction carried out at 100 °C and 130 °C during 6 hours generated solely bicyclo[4.3.0]nonane-3,8-dione (**5a**) with 50% and 69% yield, respectively (Table 1, entries 8 and 9). This product plausibly arises from a *C*-allylation step followed by an intramolecular *C*-conjugate addition/decarboxylation sequence, although throughout this study the putative intermediate **3a** proved always elusive. Using DMF and DMA as solvents at 130 °C (Table 1, entries 10 and 11), and [Pd(η^3^-C_3_H_5_)Cl]_2_, dppf] as the catalytic system in DMSO (Table 1, entry 12) did not allow further improvements for the formation of compound **5a**. Furthermore, after 1 h at 130 °C under microwave irradiation, the desired compound **5a** was isolated in 69% yield (Table 1, entry 13). Finally, separated control experiments carried out by omitting the Pd source or the ligand resulted in the exclusive recovery of the starting materials, which confirmed the need of the catalytic system for the catalytic process (Table 1, entries 14–16).

**Table 1:** Optimization of the reaction conditions.

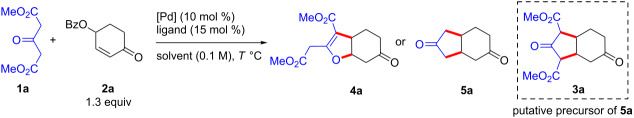					

Entry	[Pd]	Ligand^a^	Solvent	Temp (°C), time (h)	Product, yield %^b^

1	[Pd(η^3^-C_3_H_5_)Cl]_2_^c^	dppf	THF	rt, ≈1	**4a**:**5a** = 1:0, 59
2	Pd(OAc)_2_	dppb	THF	rt, ≈1	**4a**:**5a** = 1:0, 35
3	Pd(OAc)_2_	dppb	CH_3_CN	rt, ≈1	nr^d^
4	Pd(OAc)_2_	dppb	DMF	rt, ≈1	**4a**:**5a** = 1:0, 66
**5**	**Pd(OAc)_2_**	**dppb**	**DMSO**	**rt, ≈1**	**4a:5a = 1:0, 75**
6	Pd(OAc)_2_	dppe	DMSO	rt, ≈1	**4a**, traces
7	Pd(OAc)_2_	dppb	DMSO	75, ≈1	**4a**:**5a** = 1:0, 73
8	Pd(OAc)_2_	dppb	DMSO	100, 6	**4a**:**5a** = 0:1, 50
**9**	**Pd(OAc)_2_**	**dppb**	**DMSO**	**130, 6**	**4a:5a = 0:1, 69**
10	Pd(OAc)_2_	dppb	DMF	130, 6	**5a**, trace
11	Pd(OAc)_2_	dppb	DMA	130, 6	**4a**:**5a** = 0:1, 62
12	[Pd(η^3^-C_3_H_5_)Cl]_2_^c^	dppf	DMSO	130, 6	**4a**:**5a** = 0:1, 33
**13**	**Pd(OAc)_2_**	**dppb**	**DMSO**	**130 (MW, 1)**	**4a:5a = 0:1, 69**
14		dppb	DMSO	rt → 100, 6	nr^d^
15	Pd(OAc)_2_		DMSO	rt → 100, 6	nr^d^
16			DMSO	rt → 100, 6	nr^d^

^a^dppf: bis(diphenylphosphino)ferrocene, dppb: 1,4-bis(diphenylphosphino)butane, ddpe: 1,4-bis(diphenylphosphino)ethane; ^b^isolated yields after completion of **1a** monitored by TLC; ^c^5 mol %; ^d^no reaction.

The above work allowed obtaining the optimal reaction conditions for the generation of the furocycloalkanone **4a** [Pd(OAc)_2_ (10 mol %), dppb (15 mol %) in DMSO, 1 h at rt (conditions A)] as well as for the formation of the bicyclo[4.3.0]nonane-3,8-dione (**5a**) [Pd(OAc)_2_ (10 mol %), dppb (15 mol %) in DMSO at 130 °C, 6 h (conditions B), or 1 h under microwave irradiation (conditions C)].

### Scope

**Scheme 3:**
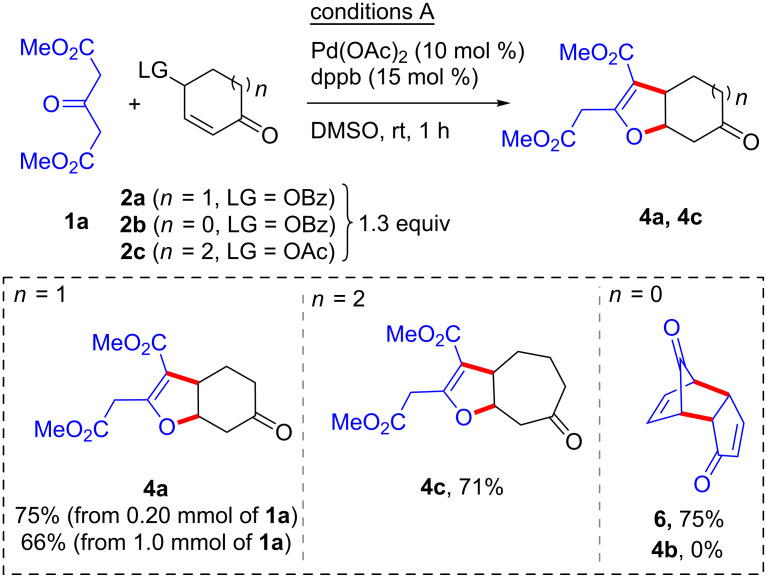
C–C/O–C bond forming annulations with dimethyl 3-oxoglutarate (**1a**).

The scope of the C–C/O–C [3 + 2] annulation between dimethyl 3-oxoglutarate (**1a**) and six- (**2a**), five- (**2b**) and seven-membered (**2c**) cyclic α,β-unsaturated-γ-oxycarbonyls was next studied (Scheme 3). Under the optimized conditions A*,* at room temperature in DMSO, the six- (**2a**) as well as the seven-membered (**2c**) bis-electrophiles reacted smoothly giving the furocycloalkanones **4a** and **4c** in good yields (Scheme 3). Furthermore, the protocol could be scaled up to 1 mmol without significant yield erosion. Treatment of cyclopentenone 4-benzoate (**2b**) under conditions A did not allow the formation of the corresponding C–C/O–C annulated product. Instead, elimination of the benzoate anion, very likely from the transiently formed η^3^-allylpalladium complex, gave cyclopentadienone, which underwent the known self-Diels–Alder cycloaddition to form dimer **6** [45].

**Scheme 4:**
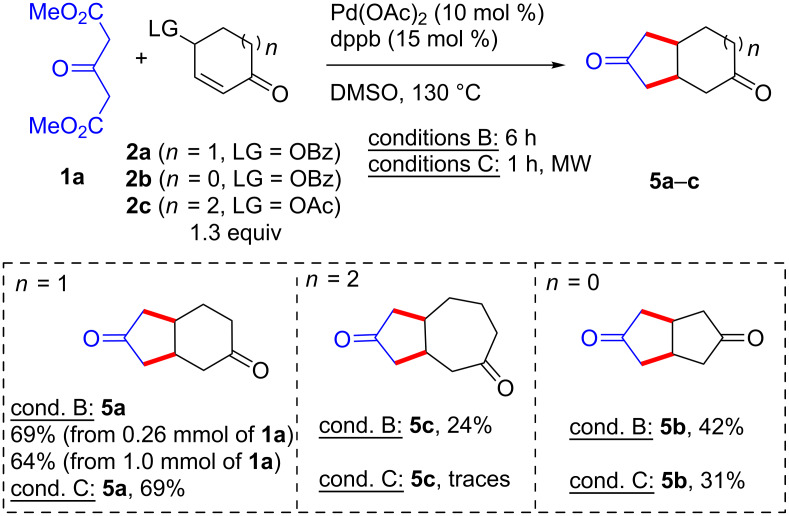
C–C/C–C bond-forming annulations with dimethyl 3-oxoglutarate (**1a**).

We next turned our attention to the [3 + 2] C–C/C–C annulation by using the conditions B and C in DMSO at 130 °C (Scheme 4). The 2-cyclohexenone 4-benzoate (**2a**) afforded the expected bicyclo[4.3.0]nonane-3,8-dione (**5a**) in 69% yield (64% from 1.0 mmol of **1a**) under either thermal conditions or microwave irradiation. Surprisingly, application of these protocols to the seven-membered bis-electrophile **2c** led to the corresponding bicyclo[5.3.0]decane-3,9-dione (**5c**) with a low yield of 24% under thermal conditions B, while microwave irradiation was ineffective. Moderate yields of bicyclo[3.3.0]octane-3,7-dione (**5b**) [46] were obtained from five-membered bis-electrophile **2b** under both conditions. This result suggests that the undesired Pd-catalyzed elimination from a γ-acyloxycyclopentenone (to give cyclopentadienone which in turn promptly dimerizes; see above), can be, at least in part, alleviated by performing the reaction at high temperature.

However, in the specific case of this latter [bis-nucleophile/bis-electrophile] couple, the desired bicyclo[3.3.0]octane-3,7-dione structure can be better obtained under basic conditions (and in the absence of Pd catalysis), as reported by Winterfeldt and Osterthun in the seventies [47].

**Scheme 5:**
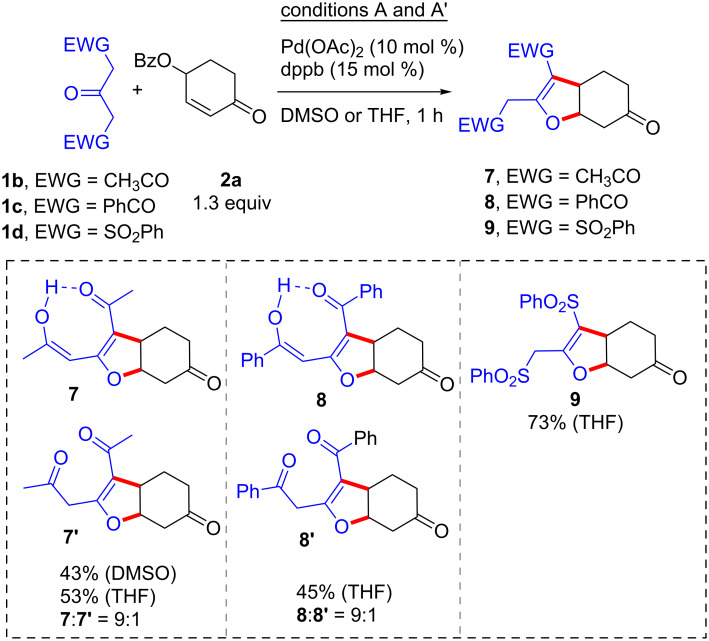
C–C/C–O bond-forming annulations with various bis-nucleophiles.

The reaction between the 1,3-activated 1,3-propanones bis-nucleophiles other than **1a** was then considered in the reaction with 2-cyclohexenone 4-benzoate (**2a**, Scheme 5). Thus, under conditions A, diacetylacetone **1b** afforded the expected product in 43% yield as a 1:9 keto/enol (**7**:**7’**) mixture. Passing from DMSO to THF (conditions A’) led to a slight increase of the yield (53%) and eased the work-up. Similarly, under conditions A', moderate to good yields of the annulated products were also obtained with dibenzoylacetone **1c** (products **8**:**8’**) and 1,3-bis-(benzenesulfonyl)propan-2-one (**1d**, product **9**), respectively. On the other hand, despite several trials using various conditions (temperature, time, and solvent) attempted conversion of the two triketones **1b**,**c** or the ketodisulfone **1d** into the corresponding 1,3-disubstitued bis-cycloalkanones **3** met always with failure.

### Mechanistic studies

**Scheme 6:**
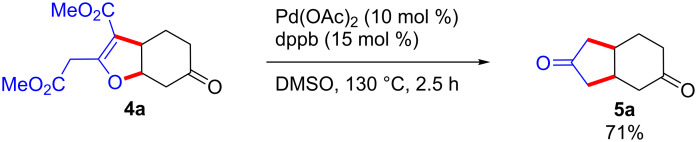
Decarboxylative rearrangement of **4a** into **5a**.

Considering that the change of reaction outcome of the dimethyl 3-oxoglutarate (**1a**) only depends on the variation of temperature in DMSO, we surmised that in the above annulation the C–C/O–C adducts **4** are the kinetic products, while the C–C/C–C adducts **5** are the thermodynamic ones. Indeed, heating compound **4a** in DMSO at 130 °C gave compound **5a** (Scheme 6). This result strongly supports the hypothesis that **4a** is the kinetic C–C/O–C annulated product, which, upon heating, rearranges to the thermodynamic C–C/C–C [3 + 2] annulated compound before undergoing two decarboxylation to give **5a**. This type of thermal 1,3-oxygen-to-carbon rearrangement was already described by Trost in the early 80’s [48–49]. In view of the high temperature needed (130 °C for several hours or under microwave irradiation), this decarboxylative rearrangement appears to require a rather high activation barrier. This activation barrier might be even greater in the case of the triketones **1b**,**c** and 1,3-bis(benzenesulfonyl)propan-2-one (**1d**), which would explain the impossibility to access the corresponding 1,3-disubstitued bis-cycloalkanone derivatives.

**Scheme 7:**
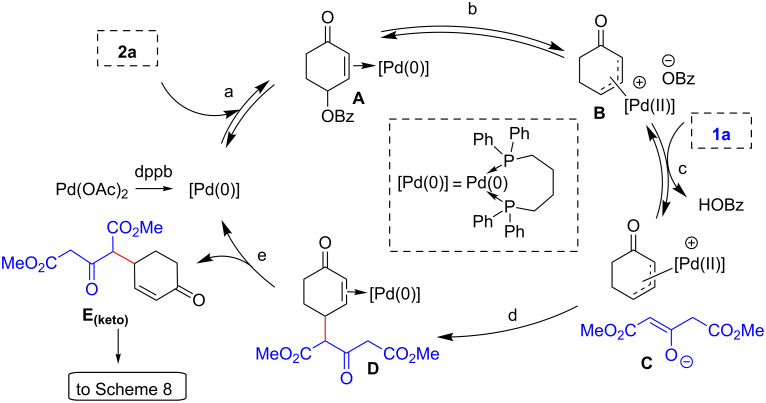
Proposed mechanism for the Pd-catalyzed part of the [3 + 2] annulation reaction.

With these considerations in mind, a plausible mechanism for this annulation is proposed for the reaction between dimethyl 3-oxoglutarate (**1a**) and 2-cyclohexenone 4-benzoate (**2a**). The reaction starts with an oxidative addition of the bis-electrophile **2a** onto the Pd(0) complex to generate η^3^-allyl complex **B** from the transient η^2^-alkene complex **A** (steps a and b). Deprotonation of the pro-nucleophile **1a** by the counter-anion of the η^3^-allyl-Pd complex exchanges the benzoate for the enolate anion (step c) [50], and following C–C bond formation from the resulting anion-scrambled complex **C** leads to the Pd(0) complex **D** (step d). Pd(0) decoordination closes the catalytic cycle delivering intermediate **E**_(keto)_ (step e) (Scheme 7).

**Scheme 8:**
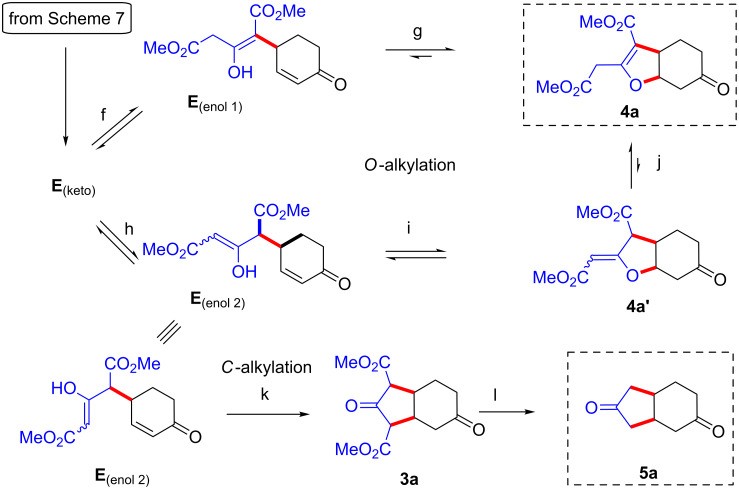
Proposed mechanism for the temperature dependent cyclization part of the [3 + 2] annulation.

The following spontaneous intramolecular *O*-conjugate addition of one of the two possible enol tautomers of **E_(keto)_** affords the kinetic C–C/O–C adduct **4a** through steps (f, g), or (h, i, j) (Scheme 8, top and middle lines) [51]. At room temperature and standard reaction times, the reaction stops at this level. However, at 130 °C the reversibility of the sequence leading to **4a** becomes important and the system has enough energy to rapidly undergo the irreversible intramolecular *C*-conjugate addition of enol **E**_(enol 2)_ followed by double decarboxylation to give the final bicyclo[4.3.0]nonane-3,8-dione (**5a**, steps k, l, Scheme 8, bottom line).

## Conclusion

In conclusion, we have successfully developed two totally selective and chemodivergent methods for the palladium-catalyzed [3 + 2] annulation between dialkyl 3-oxoglutarates and cyclic α,β-unsaturated-γ-oxycarbonyl derivatives differing simply on the variation of the reaction temperature. These new domino transformations allow a switchable (C–C/O–C to C–C/C–C) [3 + 2] annulation through an [intermolecular Pd-catalyzed *C*-allylation/intramolecular (oxygen or carbon) 1,4-conjugate addition] sequence. In particular, the conjugate addition becomes reversible if the temperature is increased, allowing to pass from the *O*-alkylation to *C*-alkylation product. Overall, the success of these [3 + 2] annulations is due to the total chemoselectivity of the initial step (*C*-allylation) as well as to the well-defined chronology of the following steps. Further work is currently ongoing to develop enantioselective versions of these new transformations.

## Experimental

**Conditions A – [3 + 2] C–C/O–C bond-forming annulations**. In a Schlenk tube, under argon atmosphere, were added Pd(OAc)_2_ (0.10 equiv), dppb (0.15 equiv) and anhydrous DMSO (0.1 M). After 10 minutes stirring, the cyclic electrophile **2a–c** (1.3 equiv) and dimethyl 3-oxoglutarate (**1a**, 1.0 equiv) were added, and the reaction was stirred at room temperature. After 1 hour stirring, the reaction mixture was filtered on a plug of silica and washed with EtOAc. The filtrate was washed with a 10% aqueous solution of NaHCO_3_. The aqueous phase was extracted with EtOAc, and the combined organic phases were washed with brine, dried over anhydrous MgSO_4_, filtered and concentrated under reduced pressure. Purification by column chromatography on silica gel afforded the corresponding product **4a** or **4c** (or compound **6** in the case of **2b**).

**Conditions B – [3 + 2] C–C/C–C bond-forming annulations***.* In a sealed tube, under argon atmosphere, were added Pd(OAc)_2_ (0.10 equiv), dppb (0.15 equiv) and anhydrous DMSO (0.1 M). After 10 min, the cyclic electrophile **2a–c** (1.3 equiv) and dimethyl 3-oxoglutarate (**1a**, 1.0 equiv) were added, and the reaction was stirred at 130 °C. After 6–8 hours stirring, the reaction mixture was filtered on a plug of silica and washed with EtOAc. The filtrate was washed with a 10% aqueous solution of NaHCO_3_. The aqueous phase was extracted with EtOAc, and the combined organic phases were washed with brine, dried over anhydrous MgSO_4_, filtered and concentrated under reduced pressure. Purification by column chromatography on silica gel afforded the corresponding product **5a**–**c**.

## Supporting Information

File 1 Full characterization of all new compounds and copies of ^1^H and ^13^C NMR spectra.
