# Supplementary material for: Switchable selectivity in Pd-catalyzed [3 + 2] annulations of γ-oxy-2-cycloalkenones with 3-oxoglutarates: C–C/C–C vs C–C/O–C bond formation
Source: Beilstein J Org Chem. 2019 May 16;15:1107–15. doi: 10.3762/bjoc.15.107 (PMC6541379; doi:10.3762/bjoc.15.107)

BEILSTEIN JOURNAL OF ORGANIC CHEMISTRY

Supporting Information

for

Switchable selectivity in Pd-catalyzed [3 + 2] annulations of γ-oxy-2-cycloalkenones with 3-oxoglutarates: C–C/C–C vs C–C/O–C bond formation

Yang Liu, Julie Oble and Giovanni Poli

Beilstein J. Org. Chem. **2019,** *15,* 1107–1115. doi:10.3762/bjoc.15.107

Full characterization of all new compounds and copies of ^{1}H and ^{13}C NMR spectra

License and Terms: This is a supporting information file under the terms of the Creative Commons Attribution License (http://creativecommons.org/licenses/by/4.0). Please note that the reuse, redistribution and reproduction in particular requires that the authors and source are credited.
The license is subject to the *Beilstein Journal of Organic Chemistry* terms and conditions: (https://www.beilstein-journals.org/bjoc)

Table of contents

I.	General information	S2
II.	Further optimizations	S3
III.	Procedures and analytical data for starting materials **1** and **2**	S4
IV.	Procedures and analytical data for bicyclic products **4**–**9**	S6
V.	^{1}H and ^{13}C NMR spectra	S12

I. General remarks

All reactions were carried out under an argon atmosphere by standard syringe and septa techniques. Glassware was flame-dried under vacuum or taken directly from the oven (100 °C) and let cool under vacuum prior to every use. Reagents and solvents were purchased from commercial sources and generally used as received. DCM, THF, CH_3CN and DMF were dried on a MBRAUN purification system MB SPS-800. Where necessary, other organic solvents or compounds were dried and/or distilled.

NMR spectra (^{1}H, ^{13}C) were recorded on a Bruker AM 300 MHz or on a Bruker AVANCE 400 MHz. NMR experiments were carried out at room temperature in deuterochloroform ($CDCl_3$). Chemical shifts are given in parts per million (ppm) using the residual non-deuterated signals as ($CDCl_3$: δ 7.26, 77.0 ppm). The terms m, s, d, t and q represent multiplet, singulet, doublet, triplet and quartet, respectively. The term (br) is used when the peak is broad, and the correct multiplicity cannot be surely assigned. Coupling constants (*J*) are given in Hertz (Hz). For previously unknown compounds, a combination of ^{13}C DEPT and 2D experiments (COSY, HSQC, HMBC) were often used to complete assignment of ^{1}H and ^{13}C signals.

IR spectra were recorded with a Tensor 27 (ATR diamond) Bruker spectrometer. IR was reported as characteristic bands (cm^{-1}). High-resolution mass spectra (HRMS) were recorded using a mass spectrometer MicroTOF from Bruker with an electron spray ion source (ESI) and a TOF detector or using a mass spectrometer from Thermo Fisher Scientific with an electron spray ion source (ESI) and a LTQ Orbitrap as detector at Institut Parisien de Chimie Moléculaire. Melting points were measured in capillary tubes on Stuart Scientific SMP3 apparatus and are uncorrected. TLC were performed on Merck 60 F254 silica gel and revealed with either a ultra-violet lamp (λ = 254 nm) or a specific color reagent (potassium permanganate, *p*-anisaldehyde, etc.). A silica gel Merck Geduran® SI 60 (40–63 µm) was used for flash column chromatography.

II. Further optimizations

Table S1: Optimization of the reaction conditions.

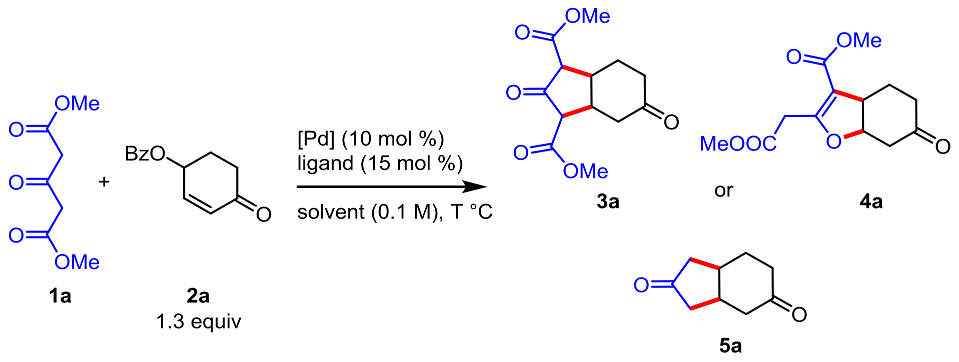

Entry	[Pd]	Ligand[a]	solvent	Temp (°C)/Time	Product, yield %[b]
1	$[Pd(\eta^3\text{-}C_3H_5)Cl]_2$[c]	dppf	THF	rt, ≈1 h	**4a**, 59
2	$[Pd(\eta^3\text{-}C_3H_5)Cl]_2$[c]	dppf	THF	70, ≈1 h	**4a**, 19
3	$Pd(OAc)_2$	dppb	THF	rt, ≈1 h	**4a**, 35
4	$Pd(OAc)_2$	dppb	CH_3CN	rt, ≈1 h	NR[d]
5	$Pd(OAc)_2$	dppb	CH_3CN	80, ≈1 h	**4a**, 30
6	$Pd(OAc)_2$	dppb	DMF	rt, ≈1 h	**4a**, 66
7	$Pd(OAc)_2$	dppb	DMSO	rt, ≈1 h	**4a**, 75
8	$Pd(OAc)_2$	dppe	DMSO	rt, ≈1 h	**4a**, trace
9	$Pd(OAc)_2$	dppb	DMSO	75, ≈1 h	**4a**, 73
10	$Pd(OAc)_2$	dppb	DMSO	100, 6 h	**5a**, 50
11	$Pd(OAc)_2$	dppb	DMSO	130, 6 h	**5a**, 69
12	$Pd(OAc)_2$	dppb	DMF	130, 6 h	**5a**, trace
13	$Pd(OAc)_2$	dppb	DMA	130, 6 h	**5a**, 62
14	$[Pd(\eta^3\text{-}C_3H_5)Cl]_2$[c]	dppf	DMSO	130, 6 h	**5a**, 33
15	$Pd(OAc)_2$	dppb	DMSO	130 (MW, 1 h)	**5a**, 69
16	$Pd(OAc)_2$	dppb	DMSO	130 (MW, 20 min)	**5a**, 35
17	$Pd(OAc)_2$	dppb	DMSO	160 (MW, 30 min)	**5a**, 60

[a]dppf: bis(diphenylphosphino)ferrocene, dppb: 1,4-bis(diphenylphosphino)butane, ddpe: 1,4-bis(diphenylphosphino)ethane; [b]Isolated yields after completion of **1a** monitored by TLC; [c]5 mol %; [d]no reaction.

III. Procedures and analytical data for starting materials 1a and 2a

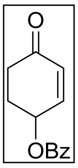

4-Oxocyclohex-2-en-1-yl benzoate (2a). Following the procedure described by Hayashi:[1] to a stirred solution of 2-cyclohexen-1-one (0.6 mL, 6.2 mmol, 1 equiv) in CH_2Cl_2 (16 mL) at 0 °C was added a solution of bromine (0.33 mL, 6.32 mmol, 1.02 equiv) in CH_2Cl_2 (16 mL) over 1 h. Et_3N (1.44 mL, 10.35 mmol, 1.7 equiv) was added and the resulting mixture was allowed to warm to room temperature and stirred for 1.5 h before it was quenched with HCl solution (1.0 M aq., 10 mL). The layers were separated and the organic layer was washed with brine (10 mL), dried ($MgSO_4$) and concentrated in vacuo to afford 2-bromo-2-cyclohexen-1-one (1.07 g, 98%).

To a solution of 2-bromo-2-cyclohexen-1-one (174 mg, 1.0 mmol, 1.0 equiv) in anhydrous acetone (5 mL), $PhCO_2Na$ (360 mg, 2.5 mmol, 2.5 equiv), 4 Å MS (348 mg, 200 wt %) and 15-crown-5 (0.56 mL, 2.8 mmol, 2.8 equiv) were added. Then, the reaction was heated at reflux for 12 hours. The mixture was filtered and the solvent was removed under reduced pressure. The residue was purified by flash chromatography on silica gel (eluent: Et_2O/pentane = 1/5; Rf 0.30 in Et_2O/pentane = 1/4) to give compound **2a** as a white solid (117 mg, 54%). These data are in good agreement with those reported in the literature.[1] **^{1}H NMR (400 MHz, $CDCl_3$):** δ 8.06 (dd, *J* = 8.5, 1.3 Hz, 2H), 7.62-7.57 (m, 1H), 7.49-7.44 (m, 2H), 6.98 (ddd, *J* = 10.2, 2.8, 1.4 Hz, 1H), 6.12 (ddd, *J* = 10.2, 1.8, 0.8 Hz, 1H), 5.86-5.80 (m, 1H), 2.74-2.66 (m, 1H), 2.58-2.45 (m, 2H), 2.31-2.21 (m, 1H).

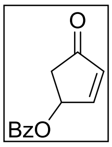

4-Benzoyloxy-2-cyclopentenone (2b). Following the procedure described by Evans:[2] furfuryl alcohol (7.35 mL, 85.0 mmol, 1 equiv) and potassium dihydrogen orthophosphate (2.1 g, 15.5 mmol, 0.18 equiv) were dissolved in water (0.5 L). The resulting solution was degassed with a stream of argon during 1 h stirring. The reaction was brought to reflux for 48 h and then cooled to room temperature. The aqueous layer was washed with EtOAc (2 x 100 mL), the combined organic layers were discarded. The aqueous layer was concentrated almost to dryness (ca. 25 mL) under reduced pressure and the residue was then thoroughly extracted with EtOAc (5 x 20 mL). The combined organic layers were dried over $MgSO_4$, filtered and the solvent removed under reduced pressure to give 4-hydroxycyclopent-2-enone as brown oil (2.943 g, 30%).

Et_3N (0.168 mL, 1.2 mmol, 1.2 equiv) and benzoic anhydride (249 mg, 1.1 mmol, 1.1 equiv) were added to a solution of the 4-hydroxycyclopent-2-enone ((98 mg, 1.0 mmol, 1.0 equiv) in dry CH_2Cl_2 (0.5 M) at 0 °C. After 10 h stirring at room temperature, the reaction was treated with a saturated aqueous $NaHCO_3$ solution, and the aqueous layer was extracted with CH_2Cl_2. The combined organic layers were washed with brine, dried over anhydrous $MgSO_4$, filtered and concentrated under reduced pressure. The crude product was purified by flash chromatography on silica gel (eluent: EtOAc/cyclohexane = 1/10; Rf 0.49 in EtOAc/cyclohexane = 1/2) to give desired product **2b** as a white

[1] (a) Hayashi, Y.; Shoji, M.; Kishida, S. *Tetrahedron Lett.* **2005**, *46*, 681. (b) Kazlauskas, R. J.; Weissfloch, A. N. E.; Rappaport, A. T.; Cuccia, L. A. *J. Org. Chem.* **1991**, *56*, 2656.

[2] (a) O'Byrne, A.; Murray, C.; Keegan, D.; Palacio, C.; Evans, P.; Morgan, B. S. *Org. Biomol. Chem.* **2010**, *8*, 539. (b) Dols, P. P. M. A.; Klunder, A. J. H.; Zwanenburg, B. *Tetrahedron* **1994**, *50*, 8515.

solid (172 mg, 85%). These data are in good agreement with those reported in the literature.[2b] **^{1}H NMR (300 MHz, $CDCl_3$):** δ 8.06-8.00 (m, 2H), 7.70 (dd, *J* = 5.7, 2.4 Hz, 1H), 7.62-7.55 (m, 1H), 7.49-7.42 (m, 2H), 6.40 (d, *J* = 5.7, 1.3 Hz, 1H), 6.14-6.08 (m, 1H), 2.95 (dd, *J* = 18.8, 6.4 Hz, 1H), 2.49 (dd, *J* = 18.8, 2.2 Hz, 1H).

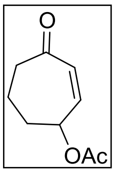

4-Acetoxy-2-cycloheptenone (2c). Following the procedure described by Nakanishi:[3] to a solution of 2-cyclohepten-1-one (80%, 275 mg, 2 mmol, 1 equiv) in carbon tetrachloride (4 mL) *N*-bromosuccinimide (500 mg, 2.8 mmol, 2.8 equiv) and benzoyl peroxide (1 mg) were added. After 3 h heating at reflux, the resulting dark brown solution was cooled to room temperature and petroleum ether (10 mL) was added to precipitate the succinimide, which was removed by filtration with additional washing with petroleum ether (5 mL). The filtrate was concentrated under reduced pressure to give a solution of crude 4-bromo-2-cycloheptenone in CCl_4 with a final volume of ca. 1 mL.

To a solution of potassium acetate (820 mg, 8.4 mmol, 4.2 equiv) and the phase transfer catalyst Aliquat 336 (55 mg, 0.14 mmol, 0.07 equiv) in H_2O (2 mL) was mixed with the previous CCl_4 solution and stirred overnight at room temperature. The mixture was diluted with 20 mL Et_2O and washed with water (2 × 5 mL) and brine. After drying the organic layer over anhydrous $MgSO_4$, the mixture was filtered and concentrated under reduced pressure. The residue was purified by flash chromatography on silica gel (eluent: EtOAc/cyclohexane = 1/8; Rf 0.22 in EtOAc/cyclohexane = 1/8) to give compound **2c** as a colorless oil (168 mg, 50%). These data are in good agreement with those reported in the literature.[3] **^{1}H NMR (400 MHz, $CDCl_3$):** δ 6.42 (dd, *J* = 12.6, 3.3 Hz, 1H), 6.01 (dd, *J* = 12.6, 2.2 Hz, 1H), 5.61-5.55 (m, 1H), 2.67-2.58 (m, 2H), 2.22-2.15 (m, 1H), 2.19 (s, 3H), 1.92-1.83 (m, 3H).

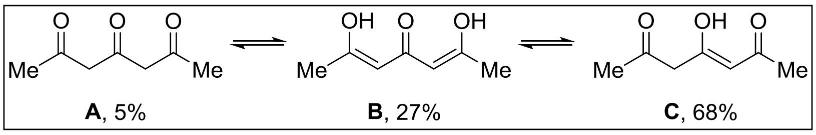

Diacetylacetone (1b). Following the procedure described by Opatz:[4] $Ba(OH)_2·8H_2O$ (10.0 g, 31.7 mmol, 2.0 equiv) was dissolved almost completely in boiling water (130 mL) under an argon atmosphere. The resulting suspension was added directly to a 50 °C warm solution of 2,6-dimethyl-γ-pyrone (1.97 g, 15.9 mmol, 1.0 equiv) in aqueous NaOH solution (8 wt %, 4 mL). A yellow precipitate formed immediately. The solution was cooled slowly to 0 °C, the crystals were filtered off, washed with aqueous NaOH solution (4 wt %), and then dissolved under ice cooling in aqueous HCl solution (15 wt %, 20 mL). The resulting solution was stirred for 1 h, and extracted with Et_2O. The combined organic layers were dried over anhydrous $MgSO_4$, filtered and concentrated under reduced pressure. The product was recrystallized from petroleum ether to afford **1b** (1.35 g, 60%) as a white solid. **^{1}H NMR (300 MHz, $CDCl_3$):** δ 15.20 (s, 1H, C), 14.17 (s, 2H, B), 5.55 (s, 1H, C),

[3] Fujimoto, Y.; Xie, R.; Tully, S. E.; Berova, N.; Nakanishi, K. *Chirality*, **2002**,*14*, 340.
[4] Schwolow, S.; Kunz, H.; Rheinheimer, J.; Opatz, T. *Eur. J. Org. Chem.* **2013**, 6519.

5.13 (s, 2H, B), 3.69 (s, 4H, A), 3.39 (s, 2H, C), 2.25 (s, 3H, C), 2.23 (s, 6H, A), 2.07 (s, 3H, C), 1.97 (s, 6H, B). These data are in good agreement with those reported in the literature.[4]

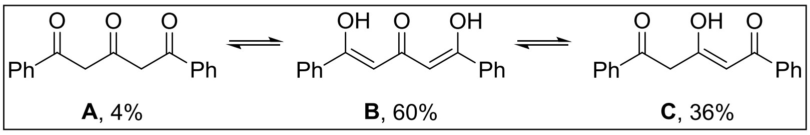

Dibenzoylacetone (1c). Following the procedure described by VanDerveer:[5] To a solution of distilled acetone (117 mg, 2.0 mmol, 1.0 equiv) in anhydrous THF (5 mL) at −78 °C was added LiHMDS (6.0 mL 6.0 mmol, 3.0 equiv, 1 mol/mL in THF) dropwise. After 15 mins, a solution of methyl benzoate (0.5 mL, 4.0 mmol, 2.0 equiv) in anhydrous THF (5 mL) was added. The mixture was stirred overnight at room temperature, and then quenched with 3 M HCl aqueous solution and extracted with Et_2O. The combined organic layers were washed with brine, dried over anhydrous $MgSO_4$, filtered and concentrated under reduced pressure. The product was recrystallized from ethanol to afford **1c** (320 mg, 60%) as a yellow solid. **^{1}H NMR (300 MHz, $CDCl_3$):** δ 15.83 (s, 1H, C), 14.76 (s, 2H, B), 8.07-7.41 (m, 30H, 10H A + 10H B + 10H C), 6.32 (s, 1H, C), 6.02 (s, 2H, B), 4.12 (s, 2H, C), 3.93 (s, 4H, A). These data are in good agreement with those reported in the literature.[5]

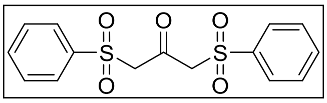

1,3-Bis-benzenesulfonylpropan-2-one (1d). Following a slightly modified version of the procedure described by Lai:[6] To a solution of sodium benzenesulfinate (1.64 g, 10.0 mmol) in CH_3CN (50mL) were added NBu_4HSO_4 (340 mg, 1.0 mmol) and 1,3-dichloropropan-2-one (635 mg, 5.0 mmol) at room temperature. The mixture was stirred at 45 °C for 24 h and then concentrated under reduced pressure. The yellow oil obtained was purified by flash chromatography on silica gel (eluent: EtOAc/cyclohexane = 1/1) to give compound **1d** (1.35 g, 80%) as a white solid. **^{1}H NMR (400 MHz, $CDCl_3$):** δ 7.88-7.82 (m, 4H), 7.73-7.68 (m, 2H), 7.60-7.55 (m, 4H), 4.51 (s, 4H). These data are in good agreement with those reported in the literature.[6]

VI. Procedures and analytical data for bicyclic products 4–9

General procedure 1 (GP1): conditions A in DMSO at rt

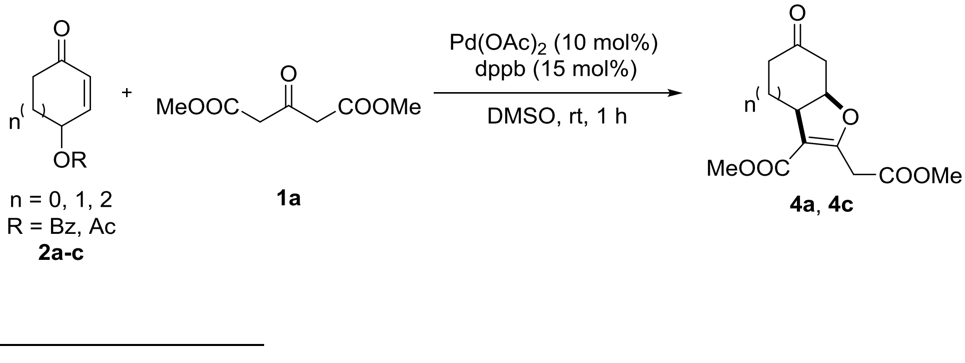

[5] Knigth, J. D.; Metz, C. R.; Beam, C. F.; Pennington, W. T.; VanDerveer, D. G. *Synth. Commun.* **2008**, *38*, 2465.
[6] Chen, Y.; Lam, Y.; Lai, Y.-H. *Org. Lett*. **2002**, *4*, 3935.

In a Schlenk tube, under an argon atmosphere, were added $Pd(OAc)_2$ (0.10 equiv), dppb (0.15 equiv) and anhydrous DMSO (0.1 M). After 10 min, the cyclic electrophilic **2a–c** (1.3 equiv) and the dimethyl 3-oxoglutarate (**1a**, 1.0 equiv) were added. The reaction was stirred at room temperature. After 1 hour, the reaction mixture was filtered on a plug of silica and washed with EtOAc. The filtrate was washed with a 10% aqueous solution of $NaHCO_3$. The aqueous phase was extracted with EtOAc, and the combined organic phases were washed with brine, dried over anhydrous $MgSO_4$, filtered and concentrated under reduced pressure. Purification by column chromatography on silica gel afforded the corresponding product **4a–c** (or compound **6** in the case of **2b**).

General procedure 2 (GP2): conditions A' in THF at rt

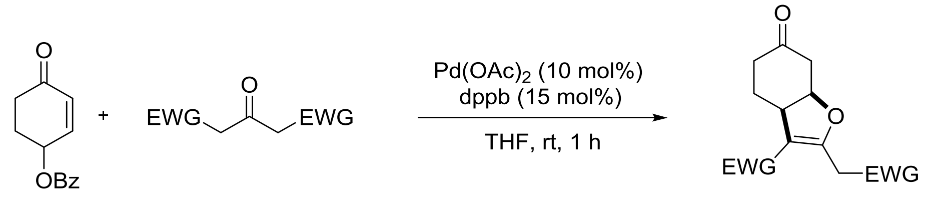

2a **1b-d** **7-9**

In a Schlenk tube, under an argon atmosphere, were added $Pd(OAc)_2$ (0.10 equiv), dppb (0.15 equiv) and anhydrous THF (0.1 M). After 10 min, the cyclic electrophilic **2a** (1.3 equiv) and the bis-nucleophile **1b–d** (1.0 equiv) were added. The reaction was stirred at room temperature. After 1 hour, the reaction mixture was filtered on a plug of silica and washed with EtOAc. The filtrate was washed with a 10% aqueous solution of $NaHCO_3$ and brine, dried over anhydrous $MgSO_4$, filtered and concentrated under reduced pressure. Purification by column chromatography on silica gel afforded the corresponding product **7–9**.

General procedure 3 (GP3): conditions B in DMSO at 130 °C

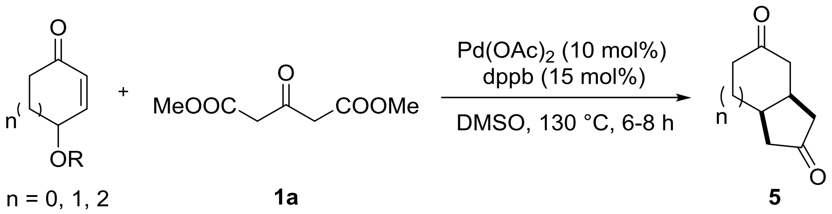

n = 0, 1, 2
R = Bz, Ac
2a-c **1a** **5**

In a sealed tube, under an argon atmosphere, were added $Pd(OAc)_2$ (0.10 equiv), dppb (0.15 equiv) and anhydrous DMSO (0.1 M). After 10 min, the cyclic electrophilic **2a-c** (1.3 equiv) and dimethyl 3-oxoglutarate (**1a**, 1.0 equiv) were added. The reaction was stirred at 130 °C. After 6 hours or 8 hours, the reaction mixture was filtered on a plug of silica and washed with EtOAc. The filtrate was washed with a 10% aqueous solution of $NaHCO_3$. The aqueous phase was extracted with EtOAc, and the combined organic phases were washed with brine, dried over anhydrous $MgSO_4$, filtered and

concentrated under reduced pressure. Purification by column chromatography on silica gel afforded the corresponding product **5a**–**c**.

General procedure 4 (GP4): conditions C in DMSO at 130 °C with microwave

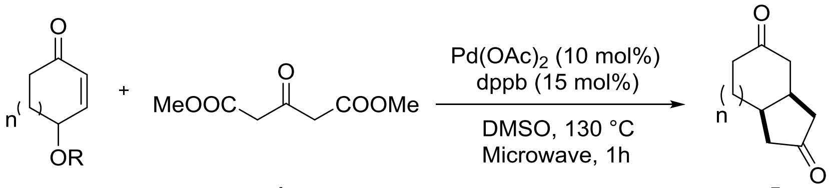

n = 0, 1, 2
R = Bz, Ac
2a-c

1a

5

In a sealed microwave tube, under an argon atmosphere, were added $Pd(OAc)_2$ (0.10 equiv), dppb (0.15 equiv) and anhydrous THF (0.1 M). After 10 mins, the cyclic electrophilic **2a–c** (1.3 equiv) and dimethyl-3-oxoglutarate (**1a**, 1.0 equiv) were added. The reaction was stirred at 130 °C under microwave irradiation conditions. After 1 hour, the reaction mixture was filtered on a plug of silica and washed with EtOAc. The filtrate was washed with a 10% aqueous solution of $NaHCO_3$. The aqueous phase was extracted with EtOAc, and the combined organic phases were washed with brine, dried over anhydrous $MgSO_4$, filtered and concentrated under reduced pressure. Purification by column chromatography on silica gel afforded the corresponding product **5a–c**.

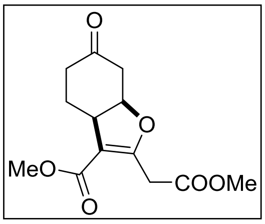

Methyl (3a*S,7a*S**)-2-(2-methoxy-2-oxoethyl)-6-oxo-3a,4,5,6,7,7a-hexahydrobenzofuran-3-carboxylate (4a).** Following **GP1** with **2a** (57 mg, 0.26 mmol) and dimethyl 3-oxoglutarate (**1a**, 29 µL, 0.20 mmol). The crude product was purified by flash chromatography on silica gel (eluent: EtOAc/cyclohexane = 1/1) to give compound **4a** (40 mg, 75%) as a white solid (or 178 mg, 66% from 1 mmol of **1a**). **Mp:** 83 °C. **IR (cm^{-1}):** 2940, 1742, 1693, 1656, 1439, 1398, 1238, 1195, 1165, 1141, 1085. **^{1}H NMR (300 MHz, $CDCl_3$):** δ 5.15 (dt, *J* = 10.0, 3.5 Hz, 1H), 3.85 (d, *J* = 16.5 Hz, 1H), 3.71 (s, 3H), 3.68 (s, 3H), 3.60-3.51 (m, 2H), 2.80 (dd, *J* = 17.1, 3.5 Hz, 1H), 2.68 (dd, *J* = 17.1, 3.5 Hz, 1H), 2.41-2.27 (m, 2H), 2.15-2.06 (m, 1H), 2.04-1.90 (m, 1H). **^{13}C NMR (75 MHz, $CDCl_3$):** δ 209.0, 168.3, 165.2, 164.2, 107.3, 81.0, 52.3, 51.1, 41.1, 39.2, 35.5, 33.8, 23.5. **HRMS (ESI)** calcd for $C_{13}H_{16}O_6Na$ $[M+Na]^+$: 291.0839; found: 291.0831.

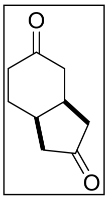

(3a*S,7aR*)-Hexahydro-1*H*-indene-2,6-dione (5a).** Following **GP3** with **2a** (57 mg, 0.26 mmol) and dimethyl 3-oxoglutarate (**1a**, 29 µL, 0.20 mmol) during 6 h. The crude product was purified by flash chromatography on silica gel (eluent: EtOAc/Cyclohexane = 2/1) to give compound **5a** (21 mg, 69%) as a colorless oil (or 97 mg, 64% from 1 mmol of **1a**).

Following **GP4** with **2a** (57 mg, 0.26 mmol) and dimethyl-3-oxoglutarate **1a** (29 µL, 0.20 mmol) during 1 h. The crude product was purified by flash chromatography on silica gel (eluent: EtOAc/Cyclohexane = 2/1) to give compound **5a** (21 mg, 69%) as a colorless oil.**IR (cm^{-1}):** 2929, 1738, 1706, 1405, 1303, 1230, 1205, 1158, 1137, 1072. **^{1}H NMR (400 MHz, $CDCl_3$):** δ 2.88-2.76 (m, 1H), 2.68-2.59 (m, 1H), 2.53 (dd, *J* = 14.7, 6.5 Hz, 1H), 2.43-2.32 (m, 4H), 2.28 (dd, *J* = 15.1, 6.2 Hz, 1H), 2.18 (dd, *J* = 18.6, 5.1 Hz, 1H), 2.10-1.97 (m, 2H), 1.81-1.68 (m, 1H). **^{13}C NMR (100 MHz, $CDCl_3$):** δ 216.9, 210.3, 43.6, 42.8, 42.6, 38.4, 37.9, 34.2, 28.0. **HRMS (ESI)** calcd for $C_9H_{12}O_2Na$ $[M+Na]^+$: 175.0730; found: 175.0723.

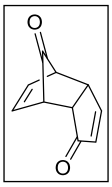

Dicyclopentadienone 6. Following **GP1** with **2b** (105 mg, 0.52 mmol) and dimethyl-3-oxoglutarate (**1a**, 58 µL, 0.40 mmol). The crude product was purified by flash chromatography on silica gel (eluent: EtOAc/Cyclohexane = 1/2) to give compound **6** (31 mg, 75%) as a white solid. **^{1}H NMR (400 MHz, $CDCl_3$):** δ 7.38 (ddd, *J* = 5.7, 2.7, 0.6 Hz, 1H), 6.37 (dd, *J* = 5.7, 1.6 Hz, 1H), 6.31 (ddd, *J* = 6.9, 3.6, 1.2 Hz, 1H), 6.17 (ddd, *J* = 6.9, 3.6, 1.2 Hz, 1H), 3.54-3.49 (m, 1H), 3.43-3.38 (m, 1H), 3.21 (ddt, *J* = 4.7, 3.6, 1.2 Hz, 1H), 2.91 (dd, *J* = 6.1, 4.9 Hz, 1H). These data are in good agreement with those reported in the literature.[7]

[7] Chaicharoenwimolkul, L.; Munmai, A.; Chairam, S.; Tewasekson, U.; Sapudom, S.; Lakliang, Y.; Somsook, E. *Tetrahedron Lett*. **2008**, *49*, 7299.

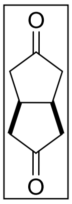

***cis*-Bicyclo[3.3.0]octane-3,7-dione (5b).** Following **GP3** with **2b** (315 mg, 1.56 mmol) and dimethyl 3-oxoglutarate (**1a**, 0.18 mL, 1.20 mmol) during 6 h. The crude product was purified by flash chromatography on silica gel (eluent: EtOAc/cyclohexane = 3/2) to give compound **5b** (70 mg, 42%) as a colorless oil. Following **GP4** with **2b** (79 mg, 0.39 mmol) and dimethyl 3-oxoglutarate (**1a**, 43 µL, 0.30 mmol) during 1 h. The crude product was purified by flash chromatography on silica gel (eluent: EtOAc/cyclohexane = 3/2) to give compound **5b** (13 mg, 31%) as a colorless oil. **^{1}H NMR (400 MHz, $CDCl_3$):** δ 3.09-2.97 (m, 2H), 2.62-2.51 (m, 4H), 2.14 (dd, *J* = 19.5, 5.3 Hz, 4H). These data are in good agreement with those reported in the literature.[8]

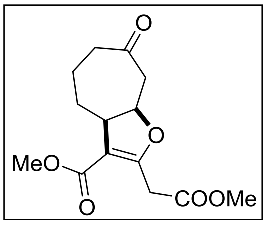

Methyl (3a*R,8a*R**)-methyl 2-(2-methoxy-2-oxoethyl)-7-oxo-4,5,6,7,8,8a-hexahydro-3a*H*-cyclohepta[*b*]furan-3-carboxylate (4c).** Following **GP2** with **2c** (66 mg, 0.39 mmol) and dimethyl 3-oxoglutarate (**1a**, 44 µL, 0.30 mmol). The crude product was purified by flash chromatography on silica gel (eluent: EtOAc/cyclohexane = 1/1) to give compound **4c** (60 mg, 71%) as a colorless oil. **IR (cm^{-1}):** 2953, 2923, 1852, 1743, 1693, 1647, 1437, 1331, 1228, 1191, 1169, 1115, 1061. **^{1}H NMR (400 MHz, $CDCl_3$):** δ 4.90 (ddd, *J* = 10.1, 8.7, 4.1 Hz, 1H), 3.74-3.67 (m, 8H), 3.40-3.33 (m, 1H), 2.99 (dd, *J* = 13.4, 8.7 Hz, 1H), 2.84 (ddd, *J* = 13.5, 4.2, 1.1 Hz, 1H), 2.59 (dddd, *J* = 12.8, 8.9, 6.3, 0.8 Hz, 1H), 2.46-2.38 (m, 1H), 2.07-1.99 (m, 1H), 1.97-1.88 (m, 1H), 1.83-1.73 (m, 1H), 1.61-1.50 (m, 1H). **^{13}C NMR (100 MHz, $CDCl_3$):** δ 207.8, 168.4, 165.3, 162.8, 108.3, 80.8, 52.3, 51.0, 45.3, 44.7, 44.6, 34.0, 26.7, 20.7. **HRMS (ESI)** calcd for $C_{14}H_{18}O_6Na$ [M+Na]$^+$: 305.0996; found: 305.0996.

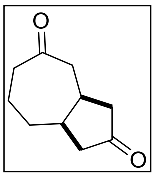

(3a*S,8a*R**)-Hexahydroazulene-2,5(1*H*,3*H*)-dione (5c).** Following **GP3** with **2c** (328 mg, 1.95 mmol) and dimethyl 3-oxoglutarate (**1a**, 0.22 mL, 1.50 mmol) during 8 hours. The crude product was purified by flash chromatography on silica gel (eluent: EtOAc/cyclohexane = 3/2) to give compound **5c** (60 mg, 24%) as a colorless oil. **IR (cm^{-1}):** 3463, 2929, 1739, 1697, 1456, 1406, 1280, 1235, 1161. **^{1}H NMR (400 MHz, $CDCl_3$):** δ 2.67-2.51 (m, 5H), 2.48-2.36 (m, 3H), 2.17-2.09 (m, 1H), 2.07-2.00 (m, 1H), 1.96-1.89 (m, 1H), 1.84-1.71 (m, 2H), 1.56-1.45 (m, 1H). **^{13}C NMR (100 MHz, $CDCl_3$):** δ 217.2, 211.8, 44.4, 44.3, 44.2, 43.4, 39.7, 35.7, 30.1, 20.4. **HRMS (ESI)** calcd for $C_{10}H_{14}O_2Na$ [M+Na]$^+$: 189.0886; found: 189.0885.

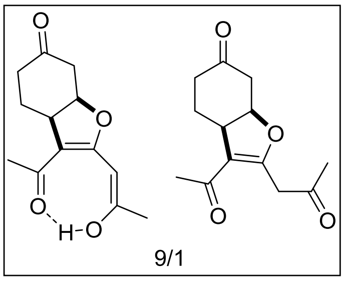

(3a*R,7a*R**)-3-Acetyl-2-(2-hydroxyprop-1-en-1-yl)-4,5,7,7a-tetrahydrobenzofuran-6(3a*H*)-one (7)** and **(3a*R**,7a*R**)-3-acetyl-2-(2-oxopropyl)-4,5,7,7a-tetrahydrobenzofuran-6(3a*H*)-one (7')**. Following **GP2** with **2a** (57 mg, 0.26 mmol) and **1b** (29 mg, 0.20 mmol). The crude product was purified by flash chromatography on silica gel (eluent: EtOAc/cyclohexane = 1/1) to give compound **7** and **7'** as a 9/1

[8] Piers, E.; Karunaratne, V. *Can. J. Chem.* **1989**, *67*, 160.

enol/ketone (25 mg, 53%) as a colorless oil. **IR (cm^{-1}):** 2925, 1718, 1585, 1381, 1237, 1223, 1135, 1067, 1043. **^{1}H NMR (300 MHz, $CDCl_3$):** δ 16.36 (s, 1H, enol), 5.41 (s, 1H, enol), 5.09 (dt, *J* = 9.7, 3.6 Hz, 2H, 1H enol + 1H ketone), 3.71 (d, *J* = 2.2 Hz, 2H, ketone), 3.63-3.54 (m, 1H, ketone), 3.49-3.40 (m, 1H, enol), 2.83 (dd, *J* = 17.1, 3.6 Hz, 2H, 1H enol + 1H ketone), 2.70 (dd, *J* = 17.1, 3.6 Hz, 2H, 1H enol + 1H ketone), 2.39-2.28 (m, 2H, 1H enol + 1H ketone), 2.27-2.14 (m, 11H, 4H enol + 7H ketone), 2.07 (s, 3H, enol), 2.04-1.96 (m, 4H, 2H enol +2H ketone). **^{13}C NMR (75 MHz, $CDCl_3$):** only enol δ 208.8, 191.7, 181.4, 167.7, 109.6, 97.4, 80.2, 41.2, 39.0, 35.7, 25.4, 23.8, 15.0. **HRMS (ESI)** calcd for $C_{13}H_{17}O_4$ $[M+H]^+$: 237.1121; found: 237.1121.

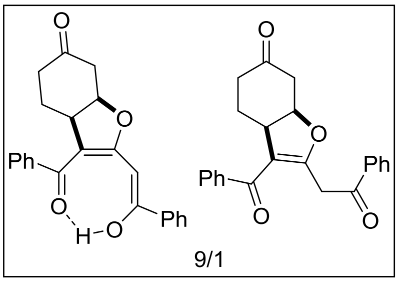

(3a*R,7a*R**)-3-Benzoyl-2-(-2-hydroxy-2-phenylvinyl)-4,5,7,7a-tetrahydrobenzofuran-6(3a*H*)-one (8)** and **3-benzoyl-2-(2-oxo-2-phenylethyl)-4,5,7,7a-tetrahydrobenzofuran-6(3a*H*)-one (8’).** Following **GP2** with **2a** (57 mg, 0.26 mmol) and **1c** (53 mg, 0.20 mmol). The crude product was purified by flash chromatography on silica gel (eluent: EtOAc/cyclohexane = 1/3) to give compound **8** and **8’** as a 90/10 enol/ketone (32 mg, 45%) as a yellow oil. **IR (cm^{-1}):** 2926, 1723, 1592, 1571, 1492, 1357, 1250, 1132, 1054. **^{1}H NMR (300 MHz, $CDCl_3$):** δ 16.41 (s, 1H, enol), 7.66-7.60 (m, 4H, 2H enol + 2H ketone), 7.55-7.50 (m, 6H, 3H enol + 3H ketone), 7.48-7.40 (m, 6H, 3H enol + 3H ketone), 7.37-7.30 (m, 4H, 2H enol + 2H ketone), 6.02 (s, 1H, enol), 5.30 (dt, *J* = 9.7, 3.6 Hz, 2H, 1H enol + 1H ketone), 3.89-3.83 (m, 2H, 1H enol + 1H ketone), 2.94 (dd, *J* = 17.1, 3.6 Hz, 2H, 1H enol + 1H ketone), 2.81 (dd, *J* = 17.1, 3.6 Hz, 2H, 1H enol + 1H ketone), 2.88-2.76 (m, 2H, ketone), 2.50-2.31 (m, 6H, 3H enol + 3H ketone), 2.23-2.13 (m, 2H, 1H enol +1H ketone). **^{13}C NMR (100 MHz, $CDCl_3$):** δ only enol 208.9, 186.2, 179.1, 165.4, 134.9, 131.6, 131.0, 130.2, 129.5 (2C), 128.4 (4C), 126.4 (2C), 113.5, 95.6, 80.5, 41.4, 41.1, 36.0, 24.0. **HRMS (ESI)** calcd for $C_{23}H_{20}O_4Na$ $[M+Na]^+$: 383.1254; found: 383.1252.

(3a*S,7a*R**)-3-(Phenylsulfonyl)-2-((phenylsulfonyl)methyl)-4,5,7,7a-tetrahydrobenzofuran-6(3a*H*)-one (9).** Following **GP2** with **2a** (57 mg, 0.26 mmol) and **1d** (68 mg, 0.20 mmol). The crude product was purified by flash chromatography on silica gel (eluent: EtOAc/cyclohexane = 3/2) to give compound **9** (63 mg, 73%) as a yellow oil.

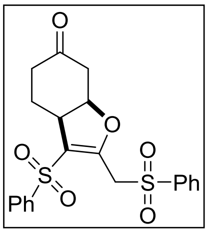

IR (cm^{-1}): 2942, 1721, 1632, 1447, 1307, 1252, 1155, 1084. **^{1}H NMR (400 MHz, $CDCl_3$):** δ 8.03-7.99 (m, 2H), 7.93-7.88 (m, 2H), 7.70-7.62 (m, 2H), 7.60-7.54 (m, 4H), 5.03 (dt, *J* = 10.3, 3.7 Hz, 1H), 4.78 (d, *J* = 14.0 Hz, 1H), 4.71 (dd, *J* = 14.0, 1.3 Hz, 1H), 3.31-3.25 (m, 1H), 2.64 (dd, *J* = 17.1, 3.6 Hz, 1H), 2.56 (dd, *J* = 17.1, 4.0 Hz, 1H), 2.24-2.17 (m, 2H), 2.08-2.01 (m, 1H), 1.88-1.78 (m, 1H). **^{13}C NMR (100 MHz, $CDCl_3$):** δ 207.3, 156.6, 140.6, 139.4, 134.2, 133.7, 129.3 (4C), 128.2 (2C), 127.6 (2C), 117.1, 81.7, 54.0, 40.8, 40.3, 35.2, 22.8. **HRMS (ESI)** calcd for $C_{21}H_{20}O_6Na$ $[M+Na]^+$: 455.0594; found: 455.0592.

IV. ^{1}H and ^{13}C NMR Spectra

4-Oxocyclohex-2-en-1-yl benzoate (2a)

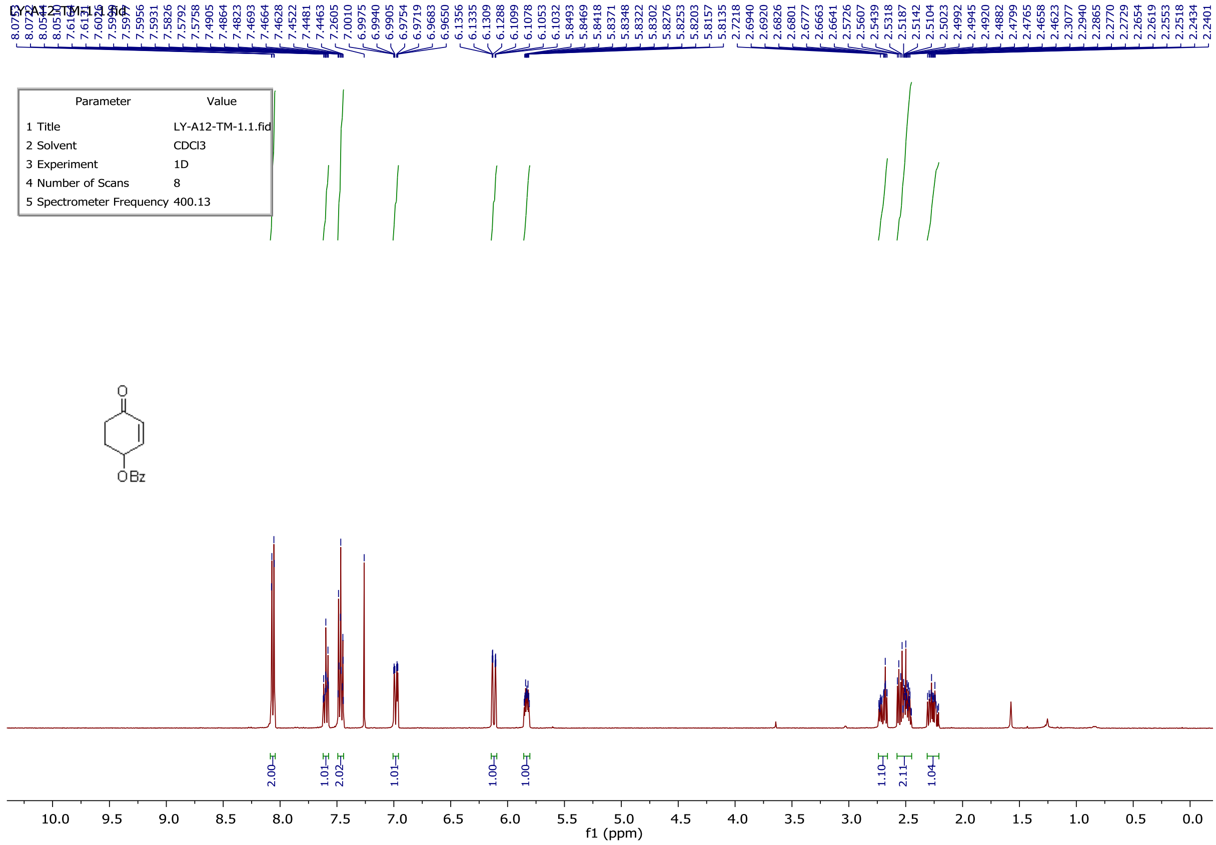

4-Benzoyloxy-2-cyclopentenone (2b)

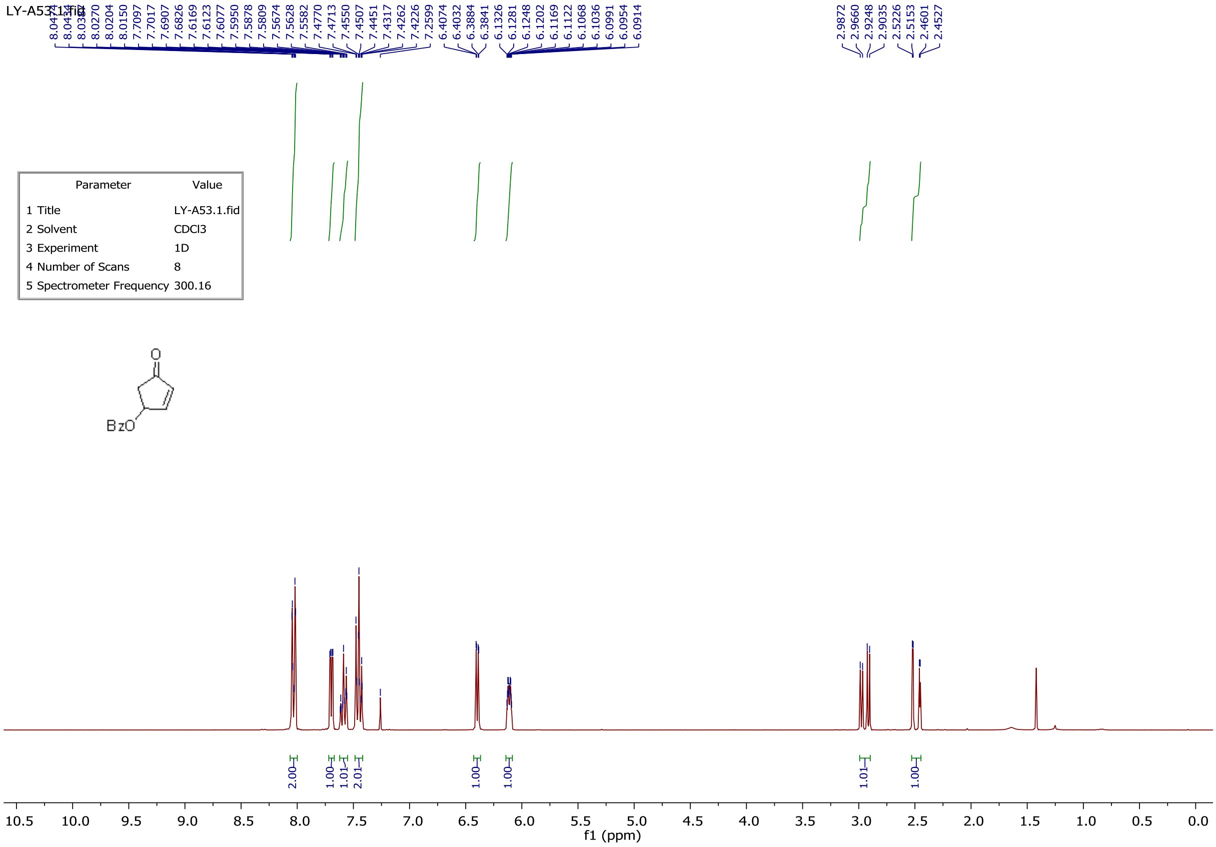

4-Acetoxy-2-cycloheptenone (2c)

LY-A152.1.fid

Parameter	Value
1 Title	LY-A152.1.fid
2 Solvent	CDCl3
3 Experiment	1D
4 Number of Scans	8
5 Spectrometer Frequency	400.13

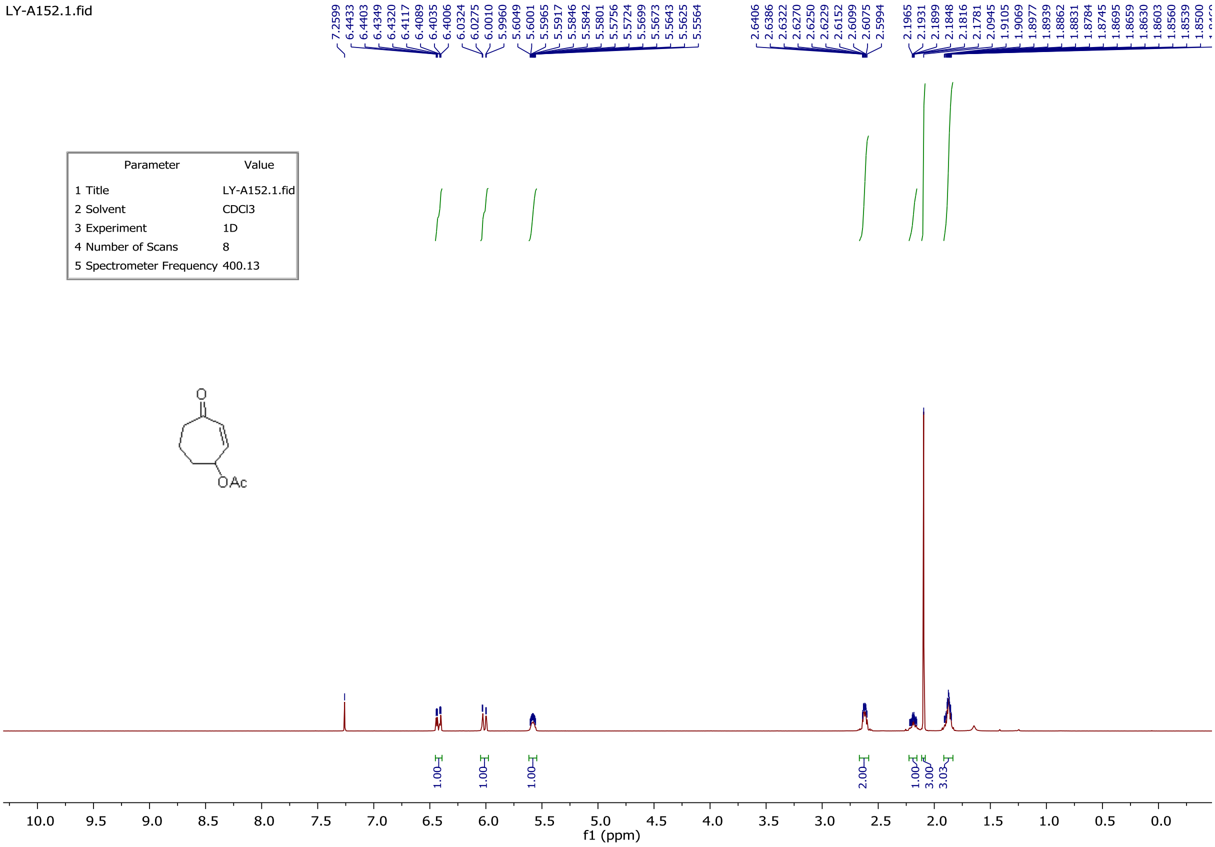

Diacetylacetone 1b

LY-C27-1/1

Parameter	Value
1 Title	LY-C27-1/ 1
2 Origin	Bruker BioSpin GmbH
3 Solvent	CDCl3
4 Experiment	1D
5 Number of Scans	8
6 Acquisition Date	2018-02-19T22:25:55
7 Modification Date	2018-02-20T09:13:44
8 Spectrometer Frequency	300.16

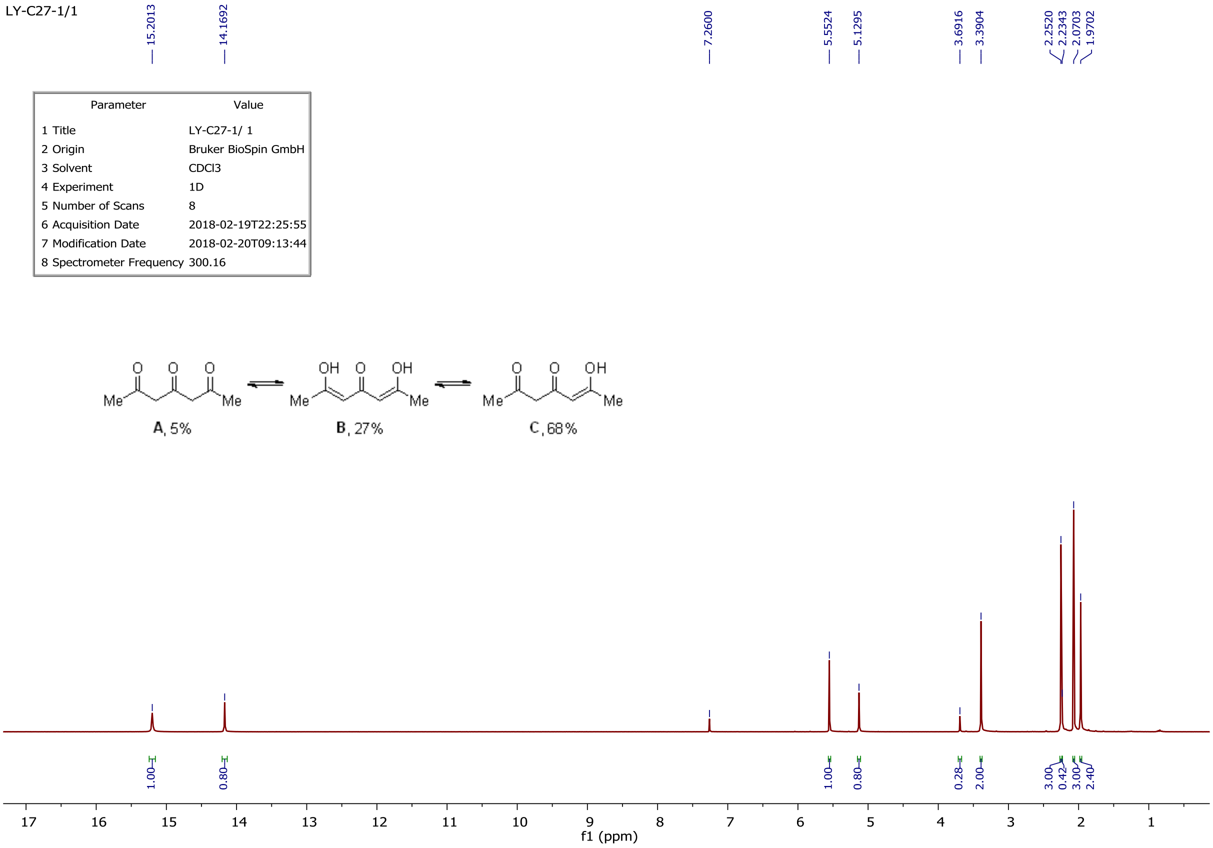

Dibenzoylacetone 1c

LY-C34/10

15.8338, 14.7639, 8.0537, 8.0500, 8.0451, 8.0363, 8.0264, 8.0210, 7.8758, 7.8706, 7.8650, 7.8496, 7.8432, 7.6097, 7.5895, 7.5850, 7.5370, 7.5261, 7.5061, 7.4980, 7.4904, 7.4850, 7.4765, 7.4651, 7.4598, 7.4515, 7.4390, 7.4297, 7.4221, 7.4157, 7.2604, 6.3188, 6.0278, 4.1195, 3.9251

Parameter	Value
1 Title	LY-C34/ 10
2 Solvent	CDCl3
3 Temperature	300.0
4 Experiment	1D
5 Number of Scans	8
6 Spectrometer Frequency	300.16

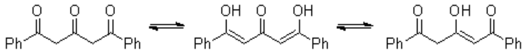

A, 4% **B**, 60% **C**, 36%

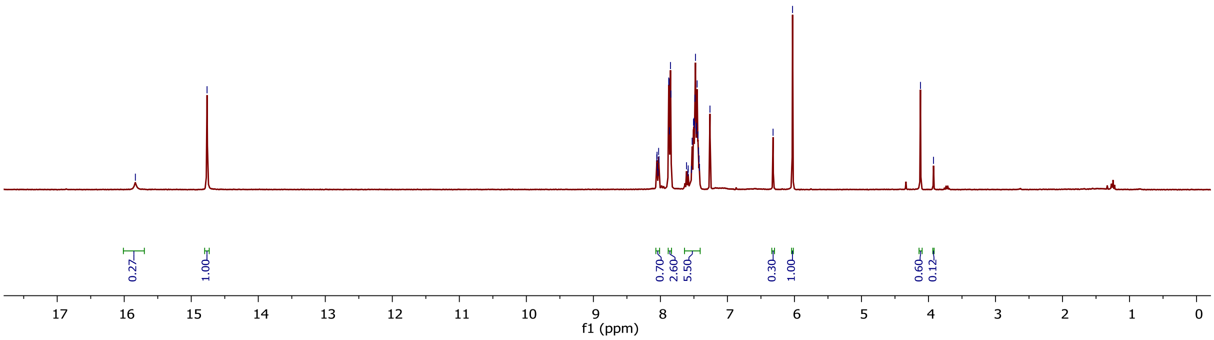

1,3-Bis-benzenesulfonylpropan-2-one (1d)

LY-C36/10

[illegible], [illegible], [illegible], [illegible], [illegible], 7.8365, 7.8352, 7.8319, 7.8296, 7.7199, 7.7028, 7.7011, 7.6993, 7.6856, 7.6824, 7.5963, 7.5951, 7.5933, 7.5911, 7.5804, 7.5782, 7.5764, 7.5746, 7.5716, 7.5581, 7.5565, 7.5548, 7.2595, 4.5061

Parameter	Value
1 Title	LY-C36/ 10
2 Origin	Bruker BioSpin GmbH
3 Solvent	CDCl3
4 Experiment	1D
5 Number of Scans	8
6 Acquisition Date	2018-03-02T10:23:29
7 Modification Date	2018-03-02T10:39:59
8 Spectrometer Frequency	400.13

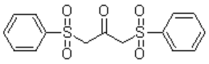

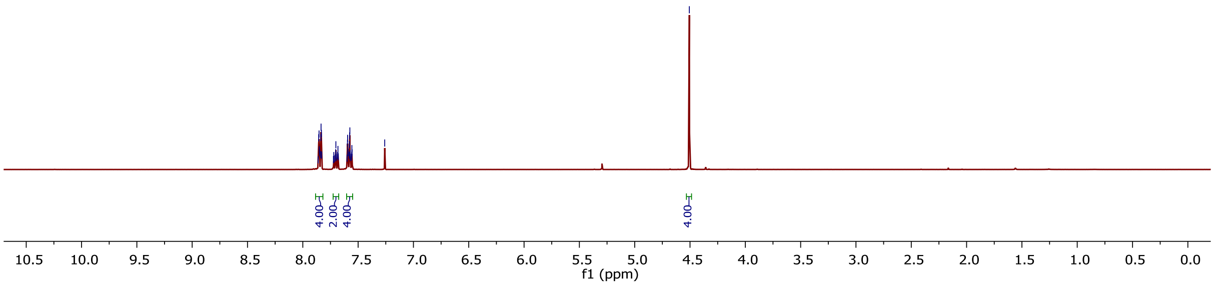

Methyl (3a*S**,7a*S**)-2-(2-methoxy-2-oxoethyl)-6-oxo-3a,4,5,6,7,7a-hexahydrobenzofuran-3-carboxylate (4a)

LY-C1-1.1.fid

7.2600, 5.1786, 5.1668, 5.1550, 5.1453, 5.1334, 5.1217, 3.8763, 3.8215, 3.7142, 3.6797, 3.5921, 3.5879, 3.5762, 3.5681, 3.5484, 3.5368, 3.5332, 3.5213, 2.8371, 2.8256, 2.7801, 2.7686, 2.7142, 2.7022, 2.6573, 2.6452, 2.4001, 2.3944, 2.3791, 2.3598, 2.3542, 2.3469, 2.3433, 2.3390, 2.3298, 2.3268, 2.2986, 2.2952, 2.2819, 2.2679, 2.1437, 2.1354, 2.1297, 2.1213, 2.1150, 2.1070, 2.0954, 2.0874, 2.0818, 2.0730, 2.0666, 2.0587, 2.0332, 2.0155, 1.9981, 1.9930, 1.9851, 1.9751, 1.9673, 1.9576, 1.9501, 1.9447, 1.9264, 1.9092

Parameter	Value
1 Title	LY-C1-1.1.fid
2 Origin	Bruker BioSpin GmbH
3 Solvent	CDCl3
4 Experiment	1D
5 Number of Scans	8
6 Acquisition Date	2018-01-15T20:06:00
7 Modification Date	2018-01-16T12:43:29
8 Spectrometer Frequency	300.16

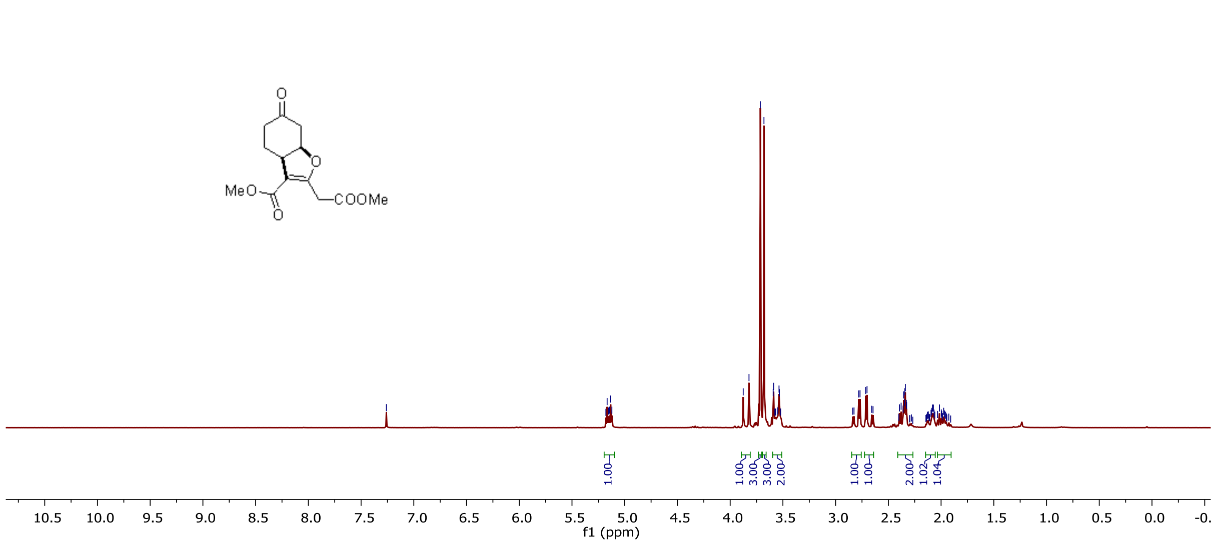

LY-C1-1.2.fid

208.9563, 168.2924, 165.2201, 164.1611, 107.3111, 80.9616, 77.4226, 76.9993, 76.5760, 52.3012, 51.1177, 41.1187, 39.2153, 35.5034, 33.8305, 23.5137

Parameter	Value
1 Title	LY-C1-1.2.fid
2 Origin	Bruker BioSpin GmbH
3 Solvent	CDCl3
4 Experiment	1D
5 Number of Scans	1024
6 Acquisition Date	2018-01-15T21:10:00
7 Modification Date	2018-01-16T12:43:31
8 Spectrometer Frequency	75.48

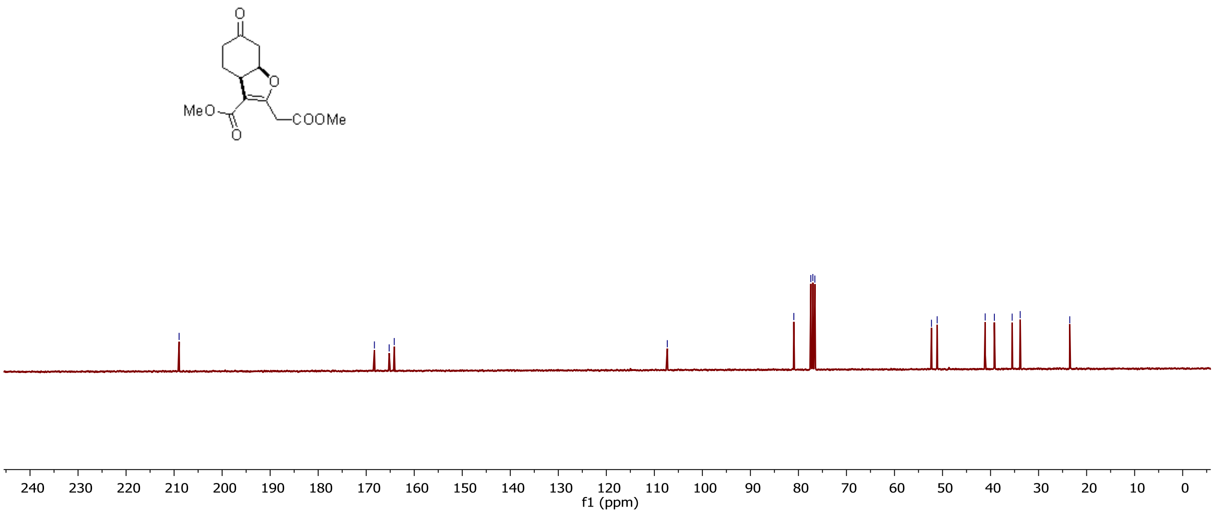

(3a*S**,7aR*)-hexahydro-1*H*-indene-2,6-dione (5a)

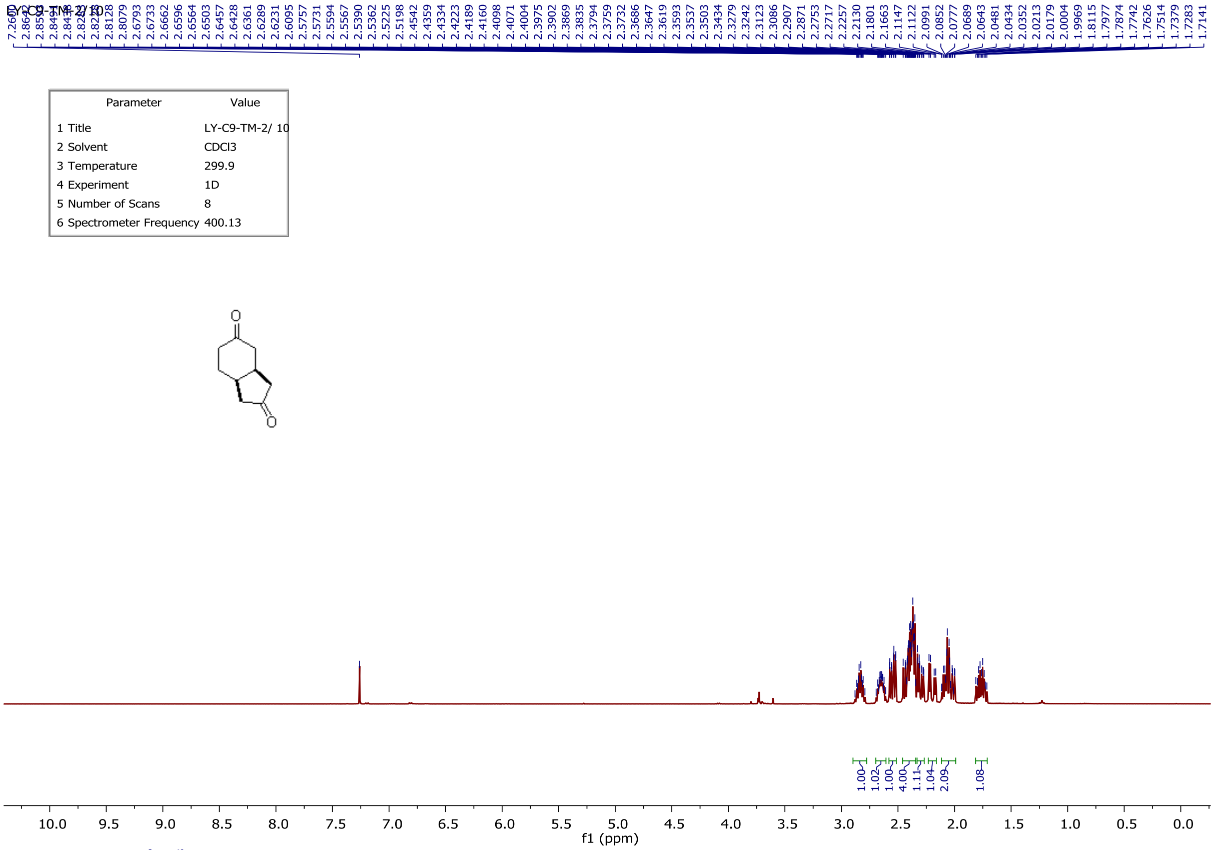

Parameter	Value
1 Title	LY-C9-TM-2/ 10
2 Solvent	CDCl3
3 Temperature	299.9
4 Experiment	1D
5 Number of Scans	8
6 Spectrometer Frequency	400.13

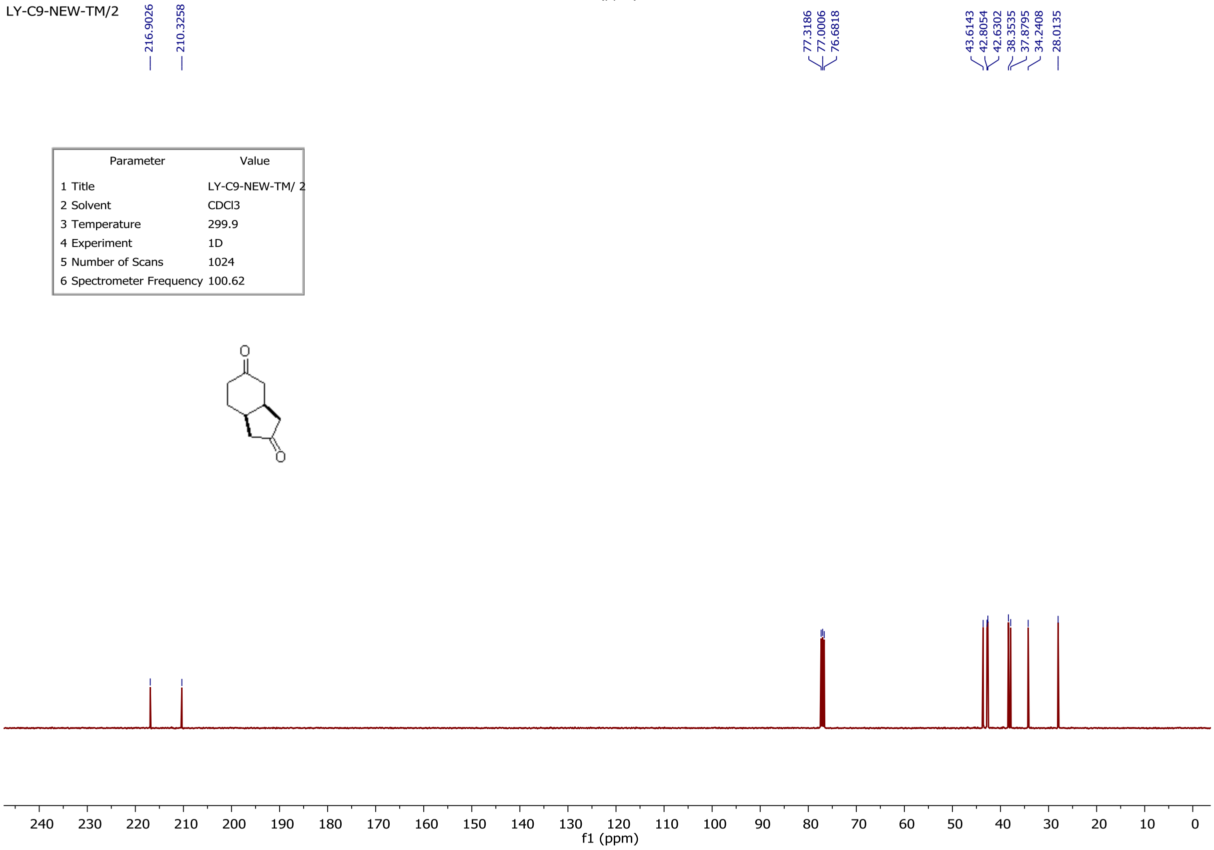

Parameter	Value
1 Title	LY-C9-NEW-TM/ 2
2 Solvent	CDCl3
3 Temperature	299.9
4 Experiment	1D
5 Number of Scans	1024
6 Spectrometer Frequency	100.62

Dicyclopentadienone 6

LY-D32/1

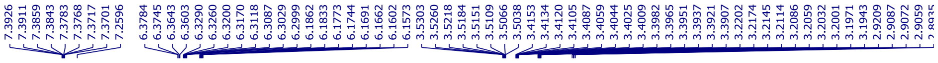

	Parameter	Value
1	Title	LY-D32/ 1
2	Solvent	CDCl3
3	Temperature	299.9
4	Experiment	1D
5	Number of Scans	8
6	Nucleus	1H

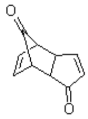

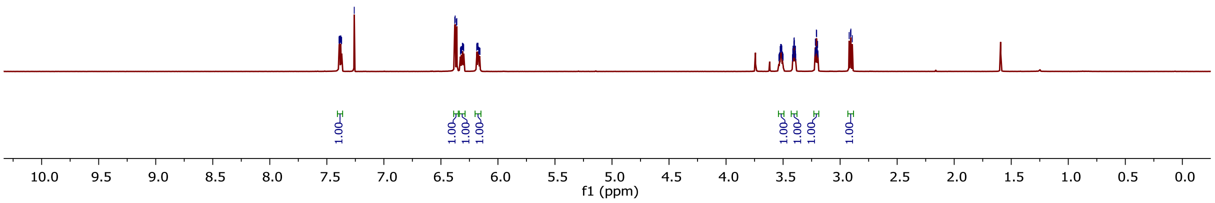

cis-Bicyclo[3.3.0]octane-3,7-dione (5b)

LY-C128/10

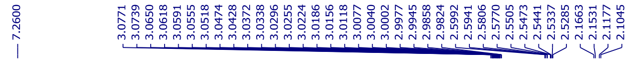

Parameter	Value
1 Title	LY-C128/ 10
2 Solvent	CDCl3
3 Temperature	299.9
4 Experiment	1D
5 Number of Scans	8
6 Spectrometer Frequency	400.13

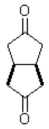

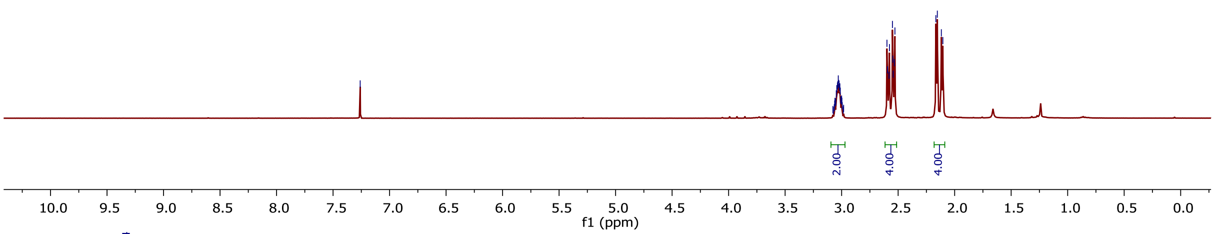

LY-C128/2

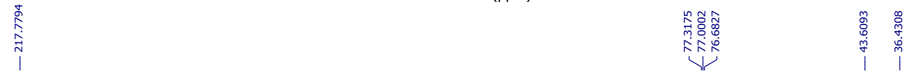

Parameter	Value
1 Title	LY-C128/ 2
2 Solvent	CDCl3
3 Temperature	299.9
4 Experiment	1D
5 Number of Scans	1024
6 Spectrometer Frequency	100.62

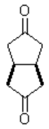

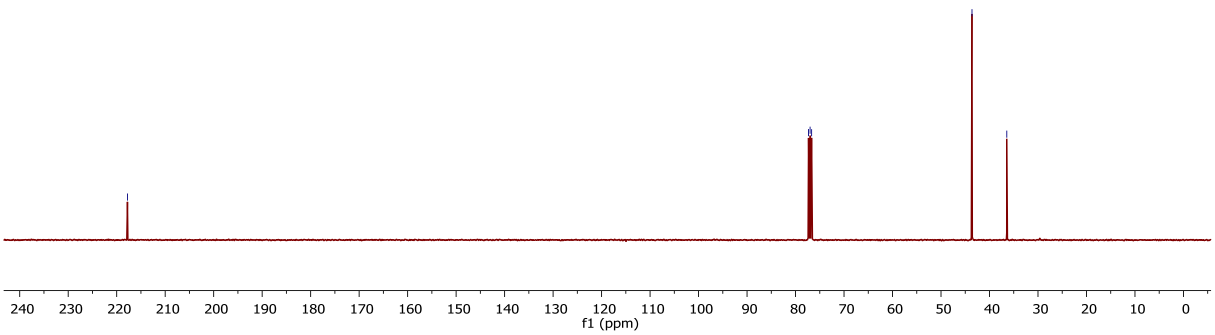

Methyl (3a*R**,8a*R**)-methyl 2-(2-methoxy-2-oxoethyl)-7-oxo-4,5,6,7,8,8a-hexahydro-3a*H*-cyclohepta[*b*]furan-3-carboxylate (4c)

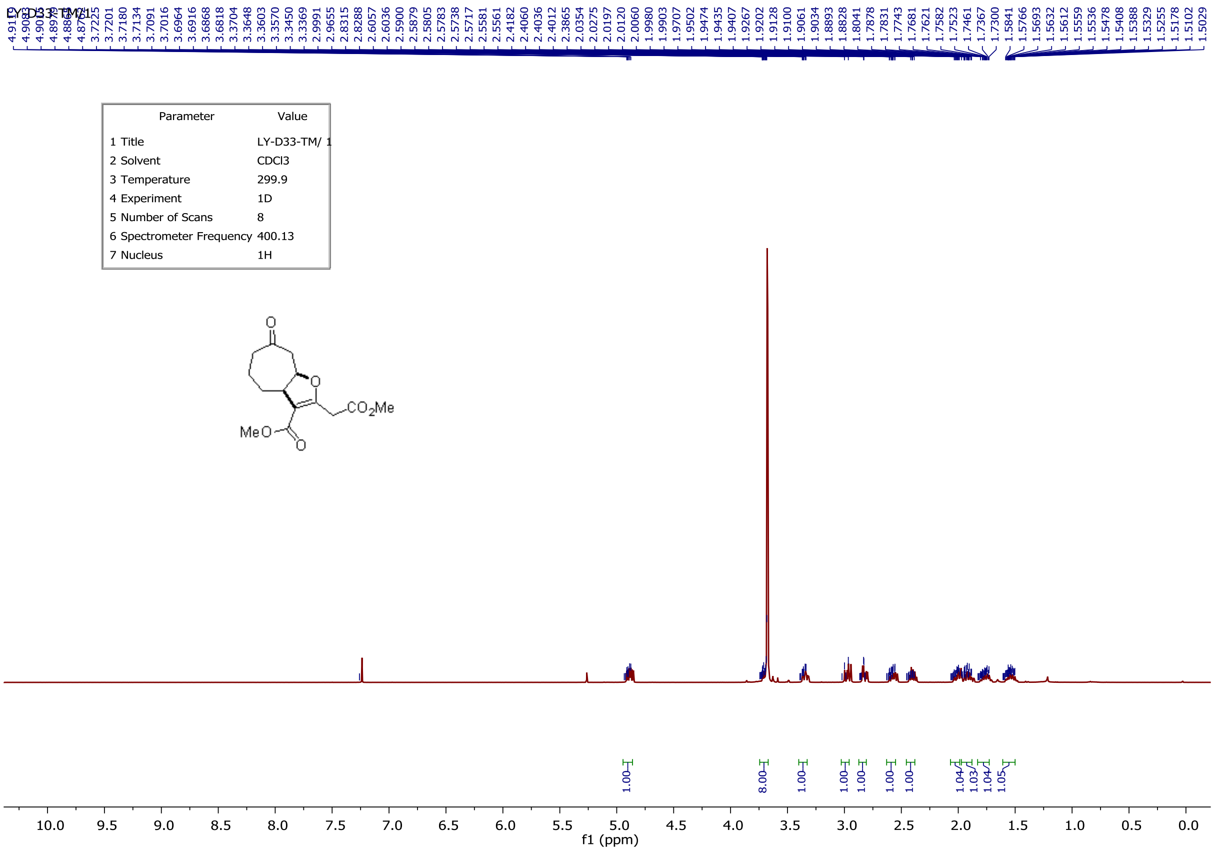

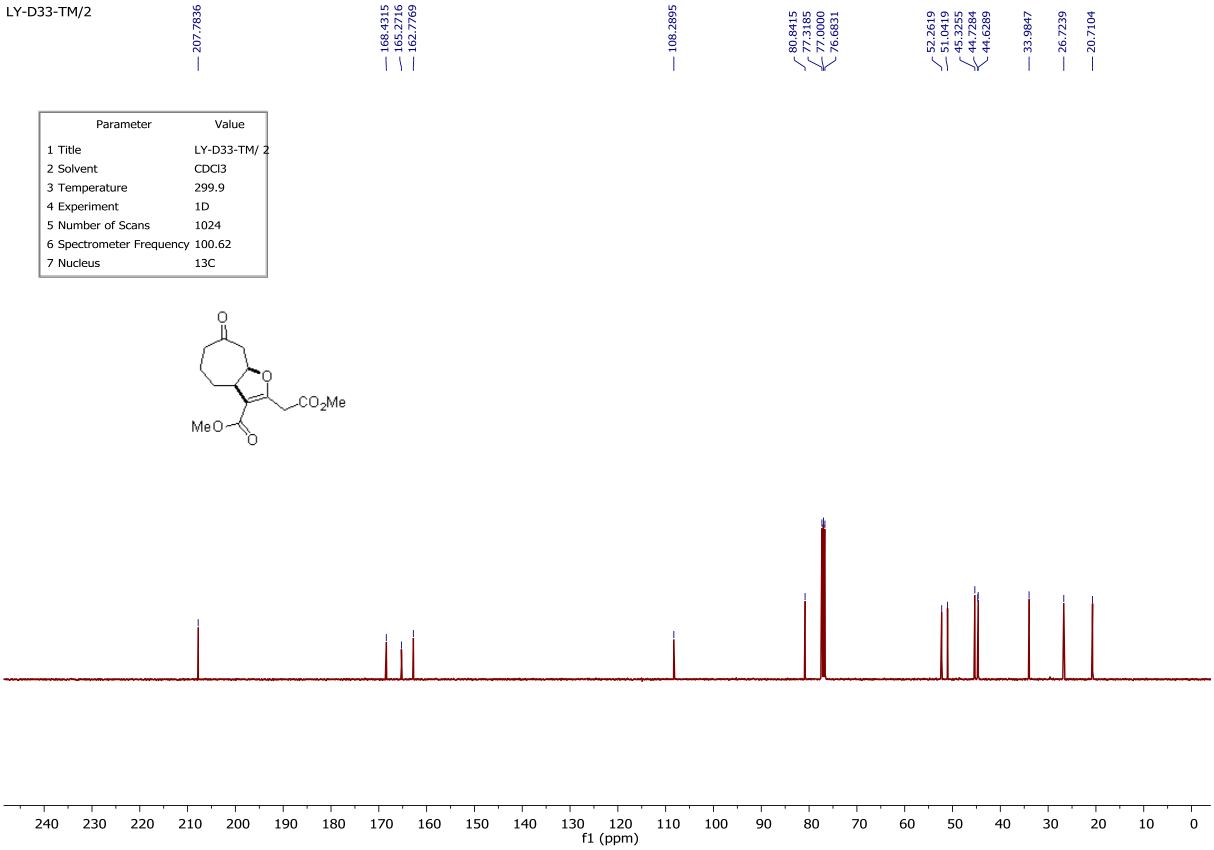

(3a*S**,8a*R**)-Hexahydroazulene-2,5(1*H*,3*H*)-dione (5c)

LY-C136-TM/1

Parameter	Value
1 Title	LY-C136-TM/ 1
2 Solvent	CDCl3
3 Temperature	299.9
4 Experiment	1D
5 Number of Scans	8
6 Spectrometer Frequency	400.13

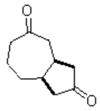

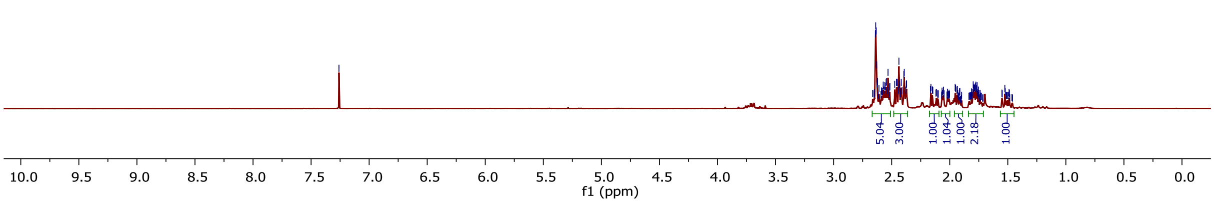

LY-C124/2

Parameter	Value
1 Title	LY-C124/ 2
2 Solvent	CDCl3
3 Temperature	299.9
4 Experiment	1D
5 Number of Scans	1024
6 Spectrometer Frequency	100.62

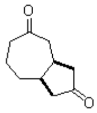

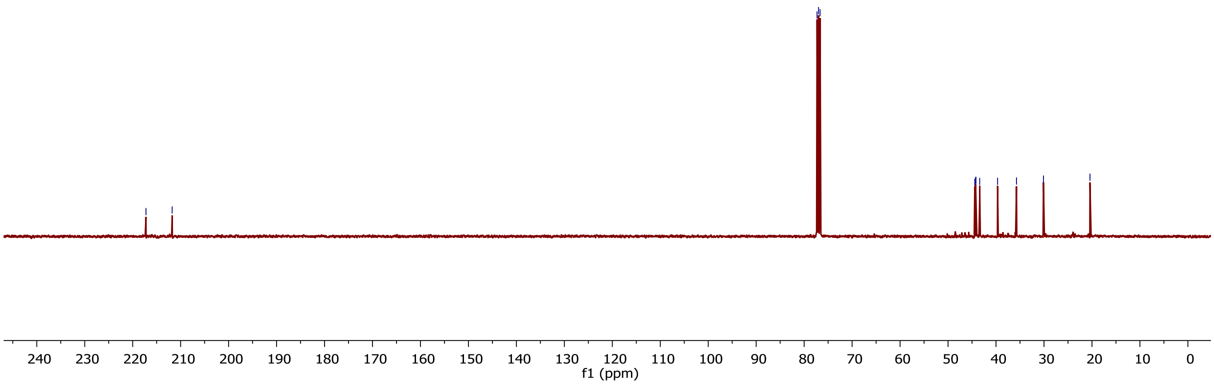

(3a*R,7a*R**)-3-Acetyl-2-(2-hydroxyprop-1-en-1-yl)-4,5,7,7a-tetrahydrobenzofuran-6(3a*H*)-one (7)** and **(3a*R**,7a*R**)-3-acetyl-2-(2-oxopropyl)-4,5,7,7a-tetrahydrobenzofuran-6(3a*H*)-one (7')**

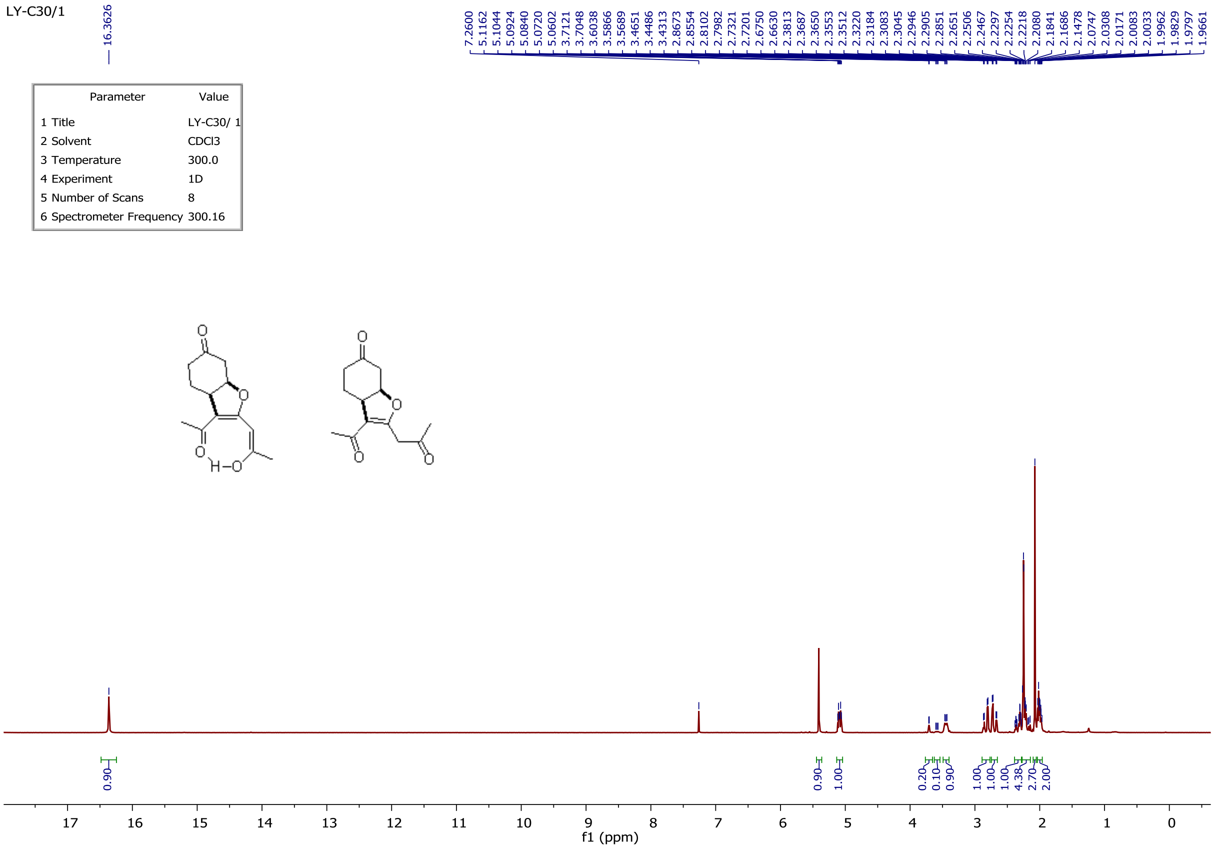

Parameter	Value
1 Title	LY-C30/ 1
2 Solvent	CDCl3
3 Temperature	300.0
4 Experiment	1D
5 Number of Scans	8
6 Spectrometer Frequency	300.16

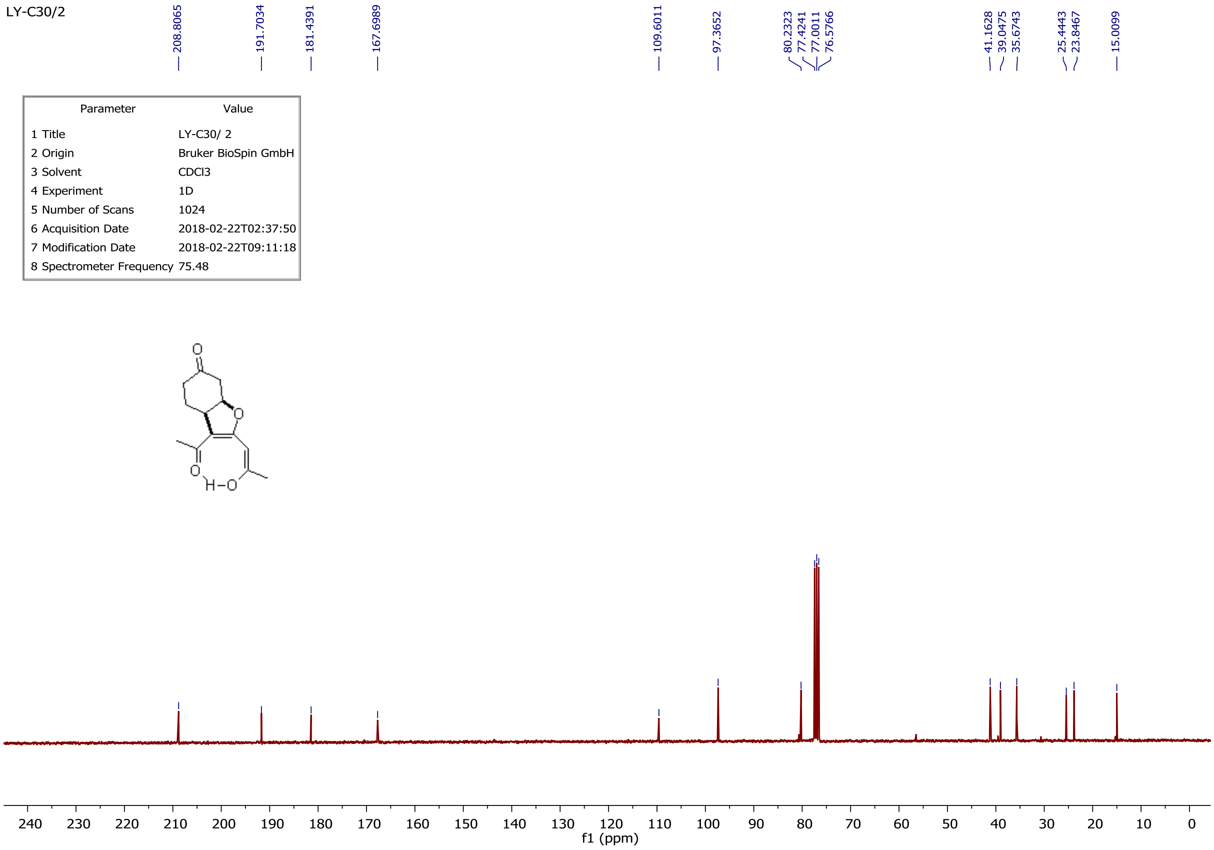

Parameter	Value
1 Title	LY-C30/ 2
2 Origin	Bruker BioSpin GmbH
3 Solvent	CDCl3
4 Experiment	1D
5 Number of Scans	1024
6 Acquisition Date	2018-02-22T02:37:50
7 Modification Date	2018-02-22T09:11:18
8 Spectrometer Frequency	75.48

(3a*R,7a*R**)-3-Benzoyl-2-(2-hydroxy-2-phenylvinyl)-4,5,7,7a-tetrahydrobenzofuran-6(3a*H*)-one (8)** and **(3a*R**,7a*R**)-3-benzoyl-2-(2-oxo-2-phenylethyl)-4,5,7,7a-tetrahydrobenzofuran-6(3a*H*)-one (8')**

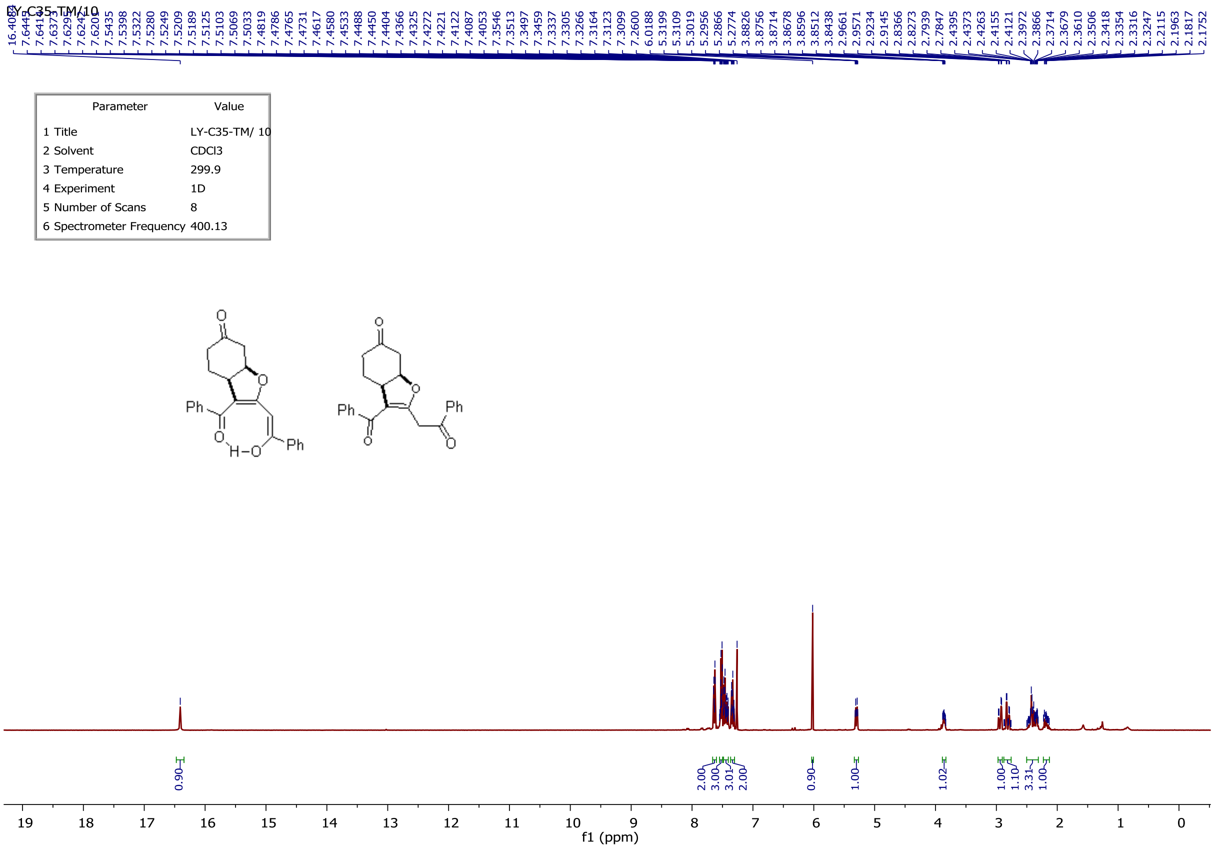

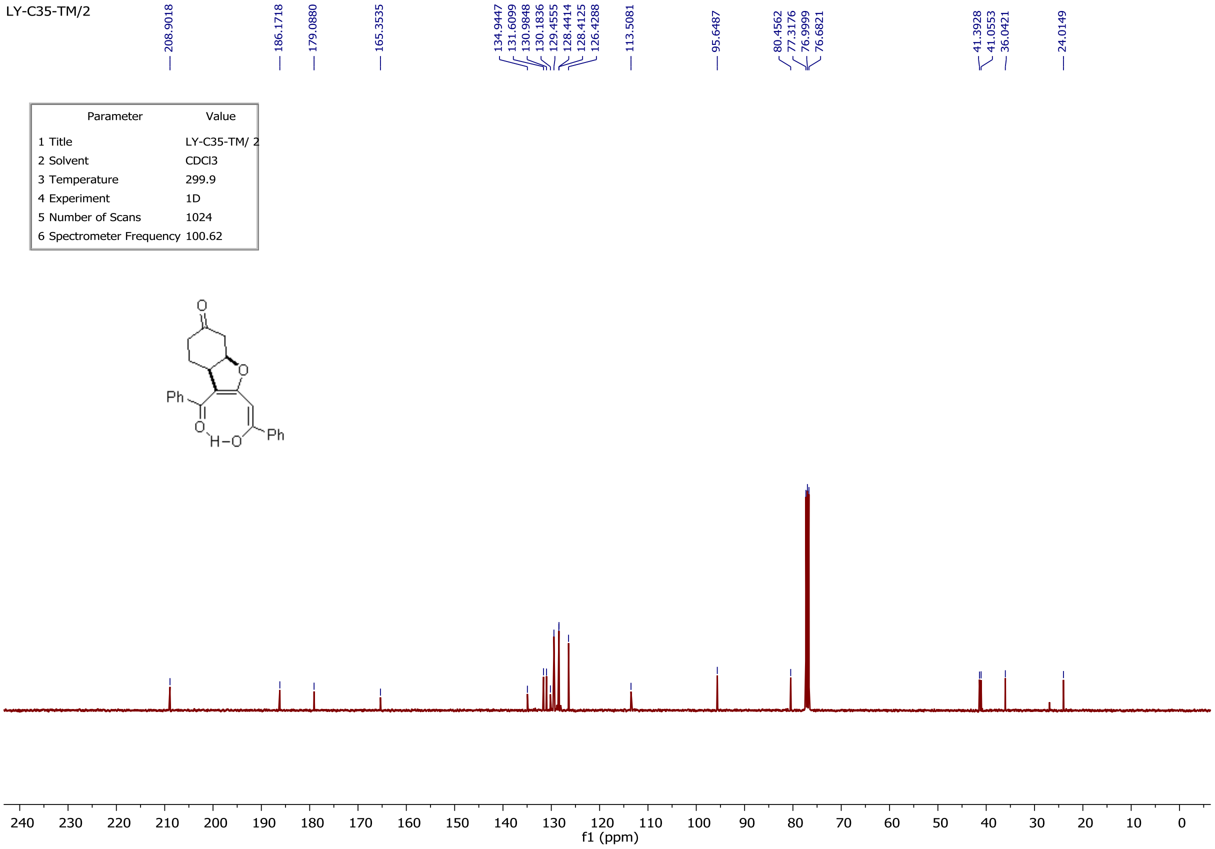

(3a*S**,7a*R**)-3-(Phenylsulfonyl)-2-((phenylsulfonyl)methyl)-4,5,7,7a-tetrahydrobenzofuran-6(3a*H*)-one (9)

LY-C37/1

8.0254, 8.0198, [illegible], [illegible], 8.0042, 7.9993, 7.9955, 7.9207, 7.9157, 7.9128, 7.9092, 7.9000, 7.8949, 7.8913, 7.6965, 7.6932, 7.6901, 7.6797, 7.6747, 7.6698, 7.6645, 7.6613, 7.6589, 7.6559, 7.6527, 7.6487, 7.6429, 7.6374, 7.6277, 7.6243, 7.6209, 7.5924, 7.5874, 7.5859, 7.5850, 7.5815, 7.5711, 7.5691, 7.5662, 7.5534, 7.5483, 7.5449, 7.5430, 7.2604, 5.0523, 5.0429, 5.0336, 5.0265, 5.0170, 5.0078, 4.7991, 4.7642, 4.7325, 4.7292, 4.6976, 4.6943, 3.2959, 3.2928, 3.2876, 3.2782, 3.2691, 3.2671, 3.2614, 2.6644, 2.6554, 2.6215, 2.6125, 2.5906, 2.5806, 2.5477, 2.5378, 2.2203, 2.2095, 2.2030, 2.2010, 2.1964, 2.1913, 2.1859, 2.0628, 2.0554, 2.0367, 2.0296, 2.0261, 2.0187, 2.0150, 1.8400, 1.8210

Parameter	Value
1 Origin	Bruker BioSpin GmbH
2 Solvent	CDCl3
3 Experiment	1D
4 Number of Scans	8
5 Relaxation Delay	1.0000
6 Spectrometer Frequency	400.13

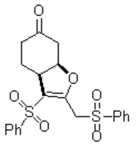

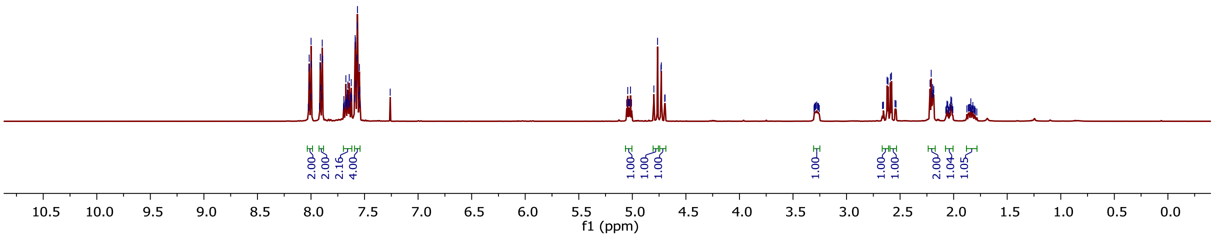

LY-C37/2

207.2859, 156.5837, 140.6028, 139.3769, 134.1951, 133.6641, 129.3439, 129.3265, 128.2117, 127.5538, 117.1272, 81.6802, 77.3179, 77.0003, 76.6829, 54.0109, 40.8374, 40.2533, 35.2000, 22.8140

Parameter	Value
1 Origin	Bruker BioSpin GmbH
2 Solvent	CDCl3
3 Experiment	1D
4 Number of Scans	1024
5 Relaxation Delay	2.0000
6 Spectrometer Frequency	100.62

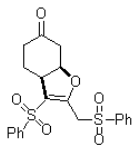

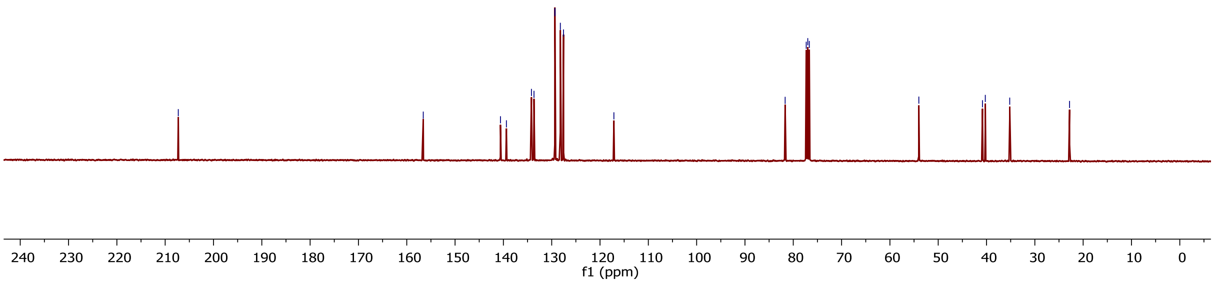

Supplement: File 1 — Full characterization of all new compounds and copies of 1H and 13C NMR spectra. [file Beilstein_J_Org_Chem-15-1107-s001.pdf]
